# TmpL, a Transmembrane Protein Required for Intracellular Redox Homeostasis and Virulence in a Plant and an Animal Fungal Pathogen

**DOI:** 10.1371/journal.ppat.1000653

**Published:** 2009-11-06

**Authors:** Kwang-Hyung Kim, Sven D. Willger, Sang-Wook Park, Srisombat Puttikamonkul, Nora Grahl, Yangrae Cho, Biswarup Mukhopadhyay, Robert A. Cramer, Christopher B. Lawrence

**Affiliations:** 1 Virginia Bioinformatics Institute and Department of Biological Sciences, Virginia Polytechnic Institute and State University, Blacksburg, Virginia, United States of America; 2 Department of Veterinary Molecular Biology, Montana State University, Bozeman, Montana, United States of America; 3 Department of Plant and Environmental Protection Sciences, University of Hawaii, Honolulu, Hawaii, United States of America; University of Melbourne, Australia

## Abstract

The regulation of intracellular levels of reactive oxygen species (ROS) is critical for developmental differentiation and virulence of many pathogenic fungi. In this report we demonstrate that a novel transmembrane protein, TmpL, is necessary for regulation of intracellular ROS levels and tolerance to external ROS, and is required for infection of plants by the necrotroph *Alternaria brassicicola* and for infection of mammals by the human pathogen *Aspergillus fumigatus*. In both fungi, *tmpL* encodes a predicted hybrid membrane protein containing an AMP-binding domain, six putative transmembrane domains, and an experimentally-validated FAD/NAD(P)-binding domain. Localization and gene expression analyses in *A. brassicicola* indicated that TmpL is associated with the Woronin body, a specialized peroxisome, and strongly expressed during conidiation and initial invasive growth *in planta*. *A. brassicicola* and *A. fumigatus ΔtmpL* strains exhibited abnormal conidiogenesis, accelerated aging, enhanced oxidative burst during conidiation, and hypersensitivity to oxidative stress when compared to wild-type or reconstituted strains. Moreover, *A. brassicicola ΔtmpL* strains, although capable of initial penetration, exhibited dramatically reduced invasive growth on Brassicas and Arabidopsis. Similarly, an *A. fumigatus ΔtmpL* mutant was dramatically less virulent than the wild-type and reconstituted strains in a murine model of invasive aspergillosis. Constitutive expression of the *A. brassicicola yap1* ortholog in an *A. brassicicola ΔtmpL* strain resulted in high expression levels of genes associated with oxidative stress tolerance. Overexpression of *yap1* in the *ΔtmpL* background complemented the majority of observed developmental phenotypic changes and partially restored virulence on plants. Yap1-GFP fusion strains utilizing the native *yap1* promoter exhibited constitutive nuclear localization in the *A. brassicicola ΔtmpL* background. Collectively, we have discovered a novel protein involved in the virulence of both plant and animal fungal pathogens. Our results strongly suggest that dysregulation of oxidative stress homeostasis in the absence of TmpL is the underpinning cause of the developmental and virulence defects observed in these studies.

## Author Summary

The critical roles of reactive oxygen species (ROS) in fungal development and virulence have been well established over the past half a century since the first experimental detection of hydrogen peroxide in fungal cells by Bach (1950). In the cell, ROS act as signaling molecules regulating physiological responses and developmental processes and are also involved in sophisticated virulence processes for many pathogenic fungi. Therefore, uncovering the biological roles of cellular ROS appears to be very important in understanding fungal development and virulence. Currently we have limited knowledge of how intracellular ROS are generated by fungal cells and which cellular ROS regulatory mechanisms are involved in establishing homeostasis. In this study we describe a novel protein, TmpL, involved in development and virulence in both plant and animal pathogenic fungi. In the absence of TmpL, dysregulation of oxidative stress homeostasis in both fungi caused developmental and virulence defects. Therefore, elucidating the role of TmpL presents an opportunity to uncover a common pathogenicity mechanism employed by both plant and animal pathogens and to develop efficient and novel therapeutics for both plant and animal fungal disease. Our findings provide new insights into mechanisms underlying the complex web of interactions between ROS and cell differentiation and the involvement of ROS for both plant and animal fungal pathogenesis.

## Introduction

Oxidative stress arises from a significant increase in the concentration of reactive oxygen species (ROS) inside the cell, and is primarily caused by either an imbalance of the cellular antioxidant capacity or a deficiency in the antioxidant system controlling ROS levels [1]. The damaging effects of ROS on DNA, proteins, lipids and other cell components and their role in pathological and aging processes is well established [2],[3],[4]. Numerous studies of pathogenic fungi have documented the crucial role of ROS produced by either fungal pathogens or their hosts in pathogenesis and defense-related activities [5],[6],[7]. There is also increasing evidence supporting an alternative view that ROS play important physiological roles as signaling molecules. ROS have been shown to be critical in immunity, cell proliferation, cell differentiation, and cell signaling pathways. However, the mechanisms by which ROS and their associated enzymes regulate development in microbial eukaryotes remain to be defined [8],[9]. Taken together, all the deleterious, pathological, and regulatory roles of ROS have generated great interest in defining the mechanisms by which ROS are produced, sensed, and managed in eukaryotes.

Because ROS readily lead to oxidative injuries, it is extremely important that the cellular ROS level be tightly controlled by complex and sophisticated redox homeostasis mechanisms. In the yeast *Saccharomyces cerevisiae*, the transcription factors Yap1 and Skn7 and a pair of related factors, Msn2 and Msn4 (Msn2/4), are implicated in controlling intracellular ROS levels [10],[11],[12]. Yap1 and Skn7 activate the expression of proteins that intercept and scavenge ROS. Yap1 is primarily controlled by a redox-sensitive nuclear export mechanism that regulates its nuclear accumulation when activated [13]. The Msn2/4 regulon contains only a small number of antioxidants but also includes heat shock proteins (HSPs), metabolic enzymes, and components of the ubiquitin-proteasome degradation pathway [14]. Recently, a heat shock transcription factor, Hsf1, has been added to the list of oxidative stress-responsive activators [15]. In addition to those found in *S. cerevisiae*, hybrid histidine kinase Mak1 and response regulator Prr1 (a Skn7 homolog), and bZIP transcription factors Atf1 and Pap1 (a Yap1 homolog) in *Schizosaccharomyces pombe* are also involved in transducing hydrogen peroxide (H_2_O_2_) signals. These proteins are required to induce catalase gene *ctt1^+^* and other genes in response to H_2_O_2_ [16],[17]. Although several similar proteins have been found and characterized in filamentous fungi, little is known about other transcriptional regulators or the defined regulatory mechanisms implicated in oxidative stress responses in filamentous fungi [7],[18],. However, orthologs of most components of the oxidative stress-sensing pathway described in yeasts are also known to be conserved in filamentous fungi such as *Aspergillus nidulans* and *Neurospora crassa* [20],[21].

Pathogenic fungi need specialized, multi-faceted mechanisms to deal with the oxidative stress encountered *in vivo* during infection. Therefore, adaptive mechanisms that confer resistance to the oxidative stress from intra- or extracellular sources may contribute to the efficient colonization and persistence of fungal pathogens in their hosts. One of the most rapid plant defense reactions encountered by plant pathogens is the so-called oxidative burst, which constitutes the production of ROS, primarily superoxide and its dismutation product, H_2_O_2_, at the site of attempted invasion [22],[23]. The ROS produced by the oxidative burst either activate plant defense responses, including programmed cell death, or function as secondary messengers in the induction of various pathogenesis-related (*PR*) genes encoding different kinds of cell wall-degrading enzymes [24],[25],[26]. Furthermore, the presence of H_2_O_2_ is essential for the formation of lignin polymer precursors via peroxidase activity, which provide additional plant barriers against pathogen attack [27].

Similarly, animal phagocytic cells produce ROS to combat invading fungal pathogens. For example, following inhalation of airborne *Aspergillus fumigatus* conidia, the normal host is protected by pulmonary innate immunity, including phagocytosis by macrophages, where the killing of the engulfed conidia is known to be directly associated with ROS production [28],[29]. *In vitro* studies of neutrophil function have shown that H_2_O_2_ effectively kills fungal hyphae [30] and that neutrophil-mediated damage is blocked by the addition of a commercial catalase [31]. Consequently, to counteract the potentially dangerous accumulation of ROS surrounding infection sites, fungal pathogens have developed diverse strategies. These include physically fortified or specialized fungal infection structures and various antioxidant defense systems through transporter-mediated effluxing, non-enzymatic antioxidants, and enzymatic scavenging systems, generally using NAD(P)H as reducing equivalents [32],[33],[34],[35].

Through a combination of computational and functional genomics approaches a novel gene *tmpL*, encoding a transmembrane protein with a N-terminal AMP-binding domain and C-terminal NAD(P)/FAD-binding domain, was characterized in this study. Previously, a protein with approximately 50% identity but lacking the AMP-binding domain present in TmpL was discovered in *A. nidulans* to be important for regulation of conidiation [36]. TmpL was initially identified during this study and referred to as the large TmpA homolog but was not functionally characterized [36]. In the present study, we characterize TmpL in both a plant and an animal fungal pathogen and provide cytochemical and genetic evidence that demonstrate a filamentous fungi-specific mechanism for control of intracellular ROS levels during conidiation and pathogenesis.

## Results

### Structure and annotation of *tmpL*

**Figure 1:**
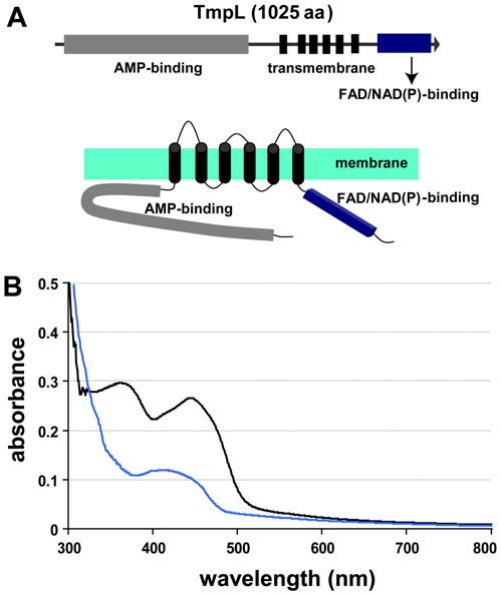
TmpL is a putative membrane flavoprotein. (**A**) Domain organization of TmpL. The predicted protein encoded by the *tmpL* gene is comprised of 1025 amino acid residues. This protein contains an AMP-binding domain, six putative transmembrane domains, and a FAD/NAD(P)-binding domain. The bottom picture shows predicted topological map of the TmpL protein. Regions of TmpL proposed to be hydrophobic membrane-spanning domains or hydrophilic domains facing the cytosol or subcompartmental matrix were identified using the TMHMM (http://www.cbs.dtu.dk/services/TMHMM-2.0/) and PRED-TMR (http://athina.biol.uoa.gr/PRED-TMR2/). (**B**) UV-visible spectra of TmpL partial recombinant protein containing FAD/NAD(P)-binding region. The absorbance spectrum shown indicates that the protein contains bound flavin (black line), demonstrating that TmpL is a FAD/NAD(P)-binding flavoprotein. Bovine serum albumin (BSA) was used as a non-flavin binding protein control (blue line). A solution of the FAD-incubated protein (2.0 mg ml^−1^) in 50 mM sodium phosphate buffer, pH 7.5, was analyzed.

Previously, seven putative nonribosomal peptide synthetase (*NPS*) genes designated as *AbNPS1* to *AbNPS7*, for *Alternaria brassicicola* nonribosomal peptide synthetase, were identified in the *A. brassicicola* genome via HMMER and BLAST analyses in our lab [37]. During this study, a *NPS*-like gene was identified with only a putative AMP-binding domain similar to an adenylation domain, followed by six transmembrane domains. There were no sequences in the adjacent region similar to thiolation and condensation domains which are typical components in the multi-modular organization of *NPS* genes. We designated this AMP-binding domain containing gene as *tmpL*, referring to the previous nomenclature but designating it as *tmpL* in lieu of large *tmpA* homolog [36]. The entire sequence of the *tmpL* gene was determined and confirmed by several sequencing events using genomic DNA and cDNA as templates for PCR based amplification and sequencing with primers based on information derived from the *A. brassicicola* genome sequence (http://www.alternaria.org). The open reading frame (ORF) of the *tmpL* is 3450 bp long and predicted to encode a protein of 1025 amino acids. The predicted TmpL hybrid protein contains an AMP-binding domain, six putative transmembrane domains, and a FAD/NAD(P)-binding domain (Figure 1A).

The *A. brassiciola* TmpL protein sequence was used to search for an *A. fumigatus* ortholog via BLASTP analysis in the genome sequence of strain CEA10. The highest sequence similarity was found for a protein encoded by a gene with the locus ID AFUB_085390. The protein sequences are 41% identical and use of protein domain prediction tools suggested that the *A. fumigatus* protein, like the *A. brassicicola* protein, has a putative N-terminal AMP-binding domain, followed by six transmembrane domains and a FAD/NAD(P)-binding domain at the C-terminus. Based on the high sequence and structural similarities to the *A. brassicicola tmpL* gene, we named this gene *A. fumigatus tmpL* as well. The ORF of the *A. fumigatus tmpL* is 3357 bp long, contains 8 predicted introns and encodes for a protein of 994 predicted amino acids.

Phylogenetic analysis indicated that TmpL and its putative orthologs are present only in filamentous fungi (Figure S1). The majority of fungal genomes shown in the phylogenetic tree contained a single putative TmpL ortholog, including *A. nidulans* that has TmpA [36]. Notable exceptions included the Basidiomycete, *Coprinus cinerea*, which contained 3, and the Sordariomycetes *Fusarium graminearum* (*Gibberella zeae*) (3), *F. oxysporum* (2), and *F. verticillioides* (2). *A. brassicicola* did not contain a putative TmpA homolog, while *A. fumigatus* contained one (EAL91362).

The AMP-binding domain of the TmpL protein showed high similarity to adenylation domains of the NPS proteins [38], which are generally involved in the activation of an amino acid substrate in the nonribosomal synthesis of polypeptides. One of the most similar sequences in the GenBank NR database was *Cochliobolus heterostrophus NPS12* (score = 2901, ID = 54%), which was reported as a putative *NPS* gene [39]. However protein functional domain searches conducted against NCBI conserved domains and the InterPro database did not detect any thiolation and condensation domains in the predicted TmpL protein. This indicates that the TmpL is indeed lacking both thiolation and condensation domains that are conserved among NPSs, and thus is not a true NPS protein.

### TmpL is a FAD/NAD(P)-binding flavoprotein

Given that TmpL does not appear to be a true NPS, we next sought to determine the function of this protein in *A. brassicicola*. The transmembrane and FAD/NAD(P)-binding domains demonstrated a high sequence similarity and predicted structure to the previously identified plasma membrane flavoprotein, TmpA, in *Aspergillus nidulans* (Figure S2) [36]. As with TmpA, the sequence analysis of the FAD/NAD(P)-binding domain showed that TmpL contains two important consensus sequences which are highly conserved in flavoproteins that bind both FAD and NAD(P). They are hypothetical FAD (RLHFD) and NAD(P) (GSGIGP) phosphate-binding domains (Figure S1), and correspond to the RXYS(T) motif for the FAD-binding domain and the GXGXXG or GT(S)G(A)IXP consensus sequences for the NAD(P)-binding domain, respectively [40],[41],[42]. In addition, protein structure homology modeling with TmpL C-terminal 247 amino acids using *Azotobacter vinelandii* NADPH:ferredoxin reductase as a template [42] via SWISS-MODEL at ExPASy (http://swissmodel.expasy.org/) showed a possible cleft formed by the two domains where the FAD and NAD(P)-binding sites were juxtaposed (data not shown). This finding was also reported in the TmpA study [36].

To support this *in silico* data, we generated a partial TmpL recombinant protein containing the FAD/NAD(P)-binding domain via *E. coli* expression. The UV-visible spectra of the partial protein observed were characteristic of a flavoprotein (Figure 1B). The absorbance peaks at 367 and 444 nm indicated that the enzyme contained bound flavin. All of these analyses suggest that TmpL possesses an enzymatic function using its FAD/NAD(P)-binding domain like other NAD(P)H-dependent flavoenzymes containing FAD or FMN cofactors such as the ferric reductase (FRE) protein group. Fungal proteins belonging to the FRE group include metalloreductase [43], NADPH-cytochrome P450 reductase [44], ferric-chelate reductase [45], and NADPH oxidases (NOX) [9].

### TmpL is associated with specific fungal Woronin bodies and shows conidial age-dependent association with peroxisomes

Next, we examined the putative subcellular localization of TmpL to gain possible insights into its cellular functions. First, *in silico* analyses were performed using wolf psort, sherloc, targetp, tmhmm, pred-tmr and signalp [46],[47],[48],[49],[50],[51]. sherloc predicted a possible subcellular localization of the TmpL protein to the peroxisomal membrane with a high probability score (0.94), while wolf psort and targetp assigned no definitive subcellular location. tmhmm and pred-tmr analyses predicted six possible transmembrane helices in TmpL similar to the results of initial protein conserved domain searches. There was no predictable N-terminal signal peptide sequence for co-translational insertion into a specific subcellular component by signalp. Taken together, these predictions indicated that TmpL might be a peroxisomal integral membrane protein with six transmembrane helices.

**Figure 2:**
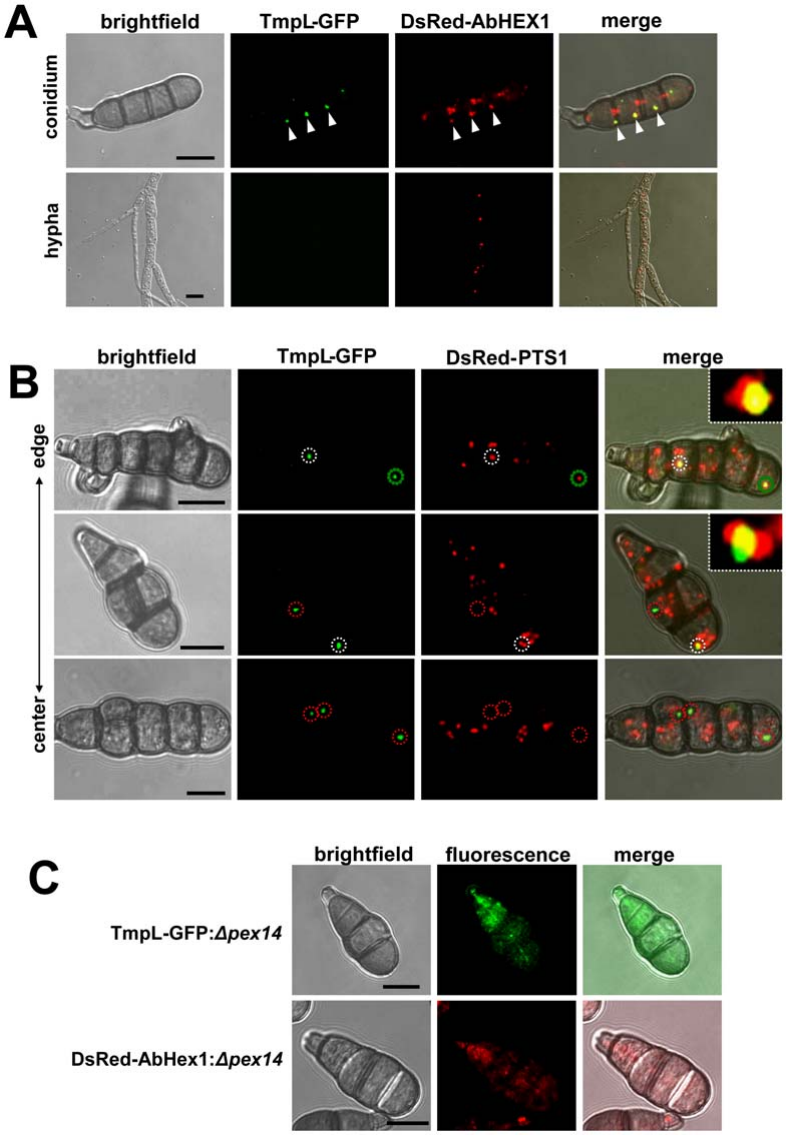
Subcellular distribution of TmpL-GFP fusion protein. (**A**) Co-localization analyses with a TmpL-GFP and DsRed-AbHex1 double-labeled strain in an *A. brassicicola* conidium (upper panel) and vegetative hypha (lower panel) were examined using confocal microscopy. TmpL-GFP localizes over cell cortex-associated or cytoplasmic DsRed-AbHex1 signals (arrowheads), not the septal pore-associated signals. Note that no TmpL-GFP signal is observed in the growing vegetative hyphae. Bars = 10 µm. (**B**) Co-localization analyses with a TmpL-GFP and DsRed-PTS1 double-labeled strain in *A. brassicicola* conidia. DsRed-PTS1 fluorescence reveals peroxisomes. A large peroxisome is completely associated with TmpL-GFP signal (a green dotted circle, top panel). The other circles denote a partial association between TmpL-GFP and DsRed-PTS1 signals (white dotted circles, top and middle panels) and a complete dissociation of TmpL-GFP with DsRed-PTS1 signals (red dotted circles, middle and bottom panels). Note that different conidial age determined by collected sites, from the center to the edge of fungal colony, shows different types of association between two fluorescence signals. Insets indicate a magnified view of each white dotted circle representing a partial association between TmpL-GFP and DsRed-PTS1 signals. Bars = 10 µm. (**C**) Localization analyses with *Δpex14* mutants on a background of either a TmpL-GFP or DsRed-AbHex1 strain. Note that the deletion of *pex14* resulted in cytoplasmic redistribution of TmpL-GFP and DsRed-AbHex1 fluorescence signals. Bars = 10 µm.

To experimentally determine the localization of TmpL within the various cell types and intracellular compartments and organelles in *A. brassicicola*, a strain expressing a TmpL-GFP fusion protein was generated. Two transformants carrying a single copy of the *tmpL*:*gfp* allele tagged at the genomic locus were identified by PCR analysis and further confirmed by Southern blot analysis (data not shown). Compared with the wild-type strain, neither of the two transformants exhibited differences in growth or pathogenesis except for expression of green fluorescence in conidia, suggesting that TmpL-GFP is fully functional. One of the transformants, A1G4, was used to analyze the localization of TmpL-GFP using confocal laser scanning fluorescence microscopy. The GFP signal was detected in conidia, but no GFP signal was detected in the vegetative mycelia of the A1G4 strain grown in complete media (CM) (Figure 2A). The GFP signals were localized in a punctate pattern in the cytoplasm as one or two tiny spots in each conidial cell, either near septae or associated with the cortical membrane. Given the previous *in silico* analyses, we hypothesized that the GFP signal might come from a specialized peroxisomal structure, the Woronin body (WB). In order to perform a co-localization test, we selected the known WB core protein HEX1 in *N. crassa*, and searched for the orthologous *abhex1* gene in *A. brassicicola*. Using the same strategy with the TmpL-GFP fusion constructs, we produced a DsRed-AbHex1 fusion protein-expressing transformant in the TmpL-GFP strain A1G4 background. DsRed-AbHex1 showed a similar punctate distribution in the cytoplasm, mostly near septal pores, but a few distant from septal pores. Figure 2A shows only DsRed-AbHex1 that are distant from septal pores co-localized with the TmpL-GFP. A separate analysis by confocal microscopy of strains that expressed either TmpL-GFP or DsRed-AbHex1 ruled out any possible cross talk between the two fluorescence signals. Although there is no literature indicating two distinct types of WBs in fungal conidia, this might suggest that TmpL is associated with a specific WB that is not associated with septal pores. Using transmission electron microscopy (TEM) of *A. brassicicola* conidia, we confirmed several WBs located distantly from septal pores (Figure S3A). As mentioned, there was no TmpL-GFP detected in vegetative hyphae, while the DsRed-AbHex1 was distributed near septal pores (Figure 2A) as reported in other studies [52],[53].

The WB has been described as evolving or being formed from peroxisome. The HEX1 assemblies emerge from the peroxisome by fission (budding off) and the nascent WB is subsequently associated with the cell cortex [54],[55]. To observe the peroxisomes and their relationship to TmpL, we co-expressed TmpL-GFP and peroxisome matrix-targeted DsRed which has a C-terminal SKL tripeptide, a peroxisome targeting signal 1 (PTS1). The TmpL-GFP was mostly associated with relatively large peroxisomes (Figure 2B). Interestingly, depending on whether conidia were harvested from the center or edge of the colony (old to young) prior to microscopic examination, three different types of association between TmpL-GFP and DsRed-PTS1 were observed. The TmpL-GFP signals in young conidia most often showed complete association with peroxisomes. Some TmpL-GFP signals mainly in older conidia were detected in a partial association with or complete dissociation from DsRed-PTS1 (Figure 2B). Together with TmpL-GFP localization with DsRed-AbHex1, these sequential associations might indicate a sequential process of WB biogenesis in *A. brassicicola*: AbHex1 assemblies in large peroxisomes (Figure 2B, a green circle), a budding event of nascent WB out of the peroxisome (Figure 2B, white circles), and a mature WB that is completely separated from the peroxisome (Figure 2B, red circles). This result was also supported by the observation of aged conidia from 21-day-old colonies, which rarely showed co-localization between TmpL-GFP and DsRed-PTS1 fusion proteins (data not shown).

It has been recently shown that PEX14, an essential component of the peroxisomal import machinery, is essential for the biogenesis of both peroxisome and WB. The deletion of *pex14* leads to complete mis-localization of peroxisomal matrix proteins containing PTS1 signal and HEX1 to the cytosol [53]. To determine whether deletion of the *A. brassicicola* homolog of *pex14* affects TmpL localization, we generated *Δpex14* mutant strains in a TmpL-GFP strain background using a linear minimal element (LME) gene disruption construct [56] and examined the mutants with confocal microscopy. In most of the TmpL-GFP:*Δpex14* mutant conidia, disruption of *pex14* resulted in an uneven distribution of the TmpL-GFP in the cytoplasm (Figure 2C). The DsRed-AbHex1:*Δpex14* mutants used as control also showed cytoplasmic distribution of the DsRed-AbHex1 as reported in the study mentioned earlier [53]. Therefore, *pex14* is related to the proper localization of TmpL protein in association with WB and peroxisome proteins governed by *pex14*-related peroxisomal import machinery, further suggesting that TmpL is associated with a specific type of WB that is not associated with septal pores.

### The organelle targeting information is located in the transmembrane region of TmpL

**Figure 3:**
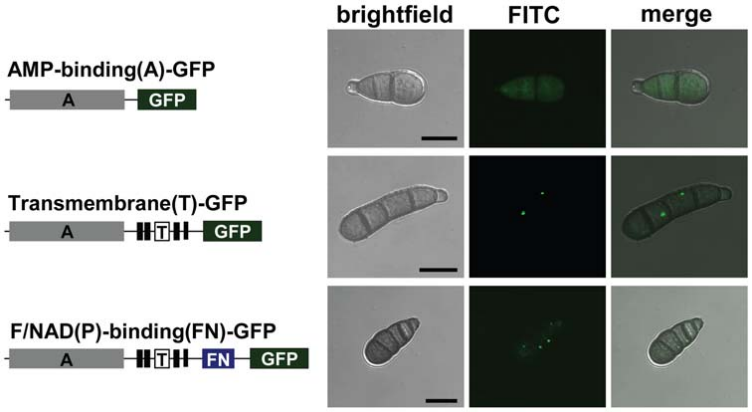
The transmembrane domain of the TmpL protein carries an organelle targeting signal. Organelle targeting of partial or complete TmpL-GFP fusion proteins. At left schematic representations: gray boxes represent the AMP-binding (A) domain of TmpL protein; six tandem black boxes represent the transmembrane (T) domain; and a blue box represents the FAD/NAD(P)-binding (FN) domain. Right micrographs show GFP signal localization patterns of each fusion protein in *A. brassicicola* conidia. Bars = 10 µm.

HEX1 and its orthologs in filamentous fungi possess a PTS1 at their C-terminal end that target it to the peroxisomal matrix [57]. However, as other known peroxisomal membrane proteins, the predicted TmpL sequences do not carry any defined localization signal peptides or PTS peptides. To identify the organelle targeting information in TmpL, we produced three transformants by appending GFP marker protein at three locations of TmpL: the AMP-binding domain, transmembrane domain, and FAD and NAD(P)-binding domain. This produced truncated TmpL-GFP fusion proteins under the control of the wild-type *tmpL* promoter (Figure 3). Using each construct, we generated three different GFP-tagged strains and examined their localization pattern. The AMP-binding-GFP fusion protein resulted in cytoplasmic distribution of the GFP signal, while the transmembrane- and FAD and NAD(P)-binding-GFP fusion proteins were concentrated in a punctate pattern in the cytoplasm (Figure 3). This suggests that the transmembrane domain carries the targeting signal to the organelle membrane.

### *tmpL* is strongly expressed during conidiation and initial invasive growth *in planta*

**Figure 4:**
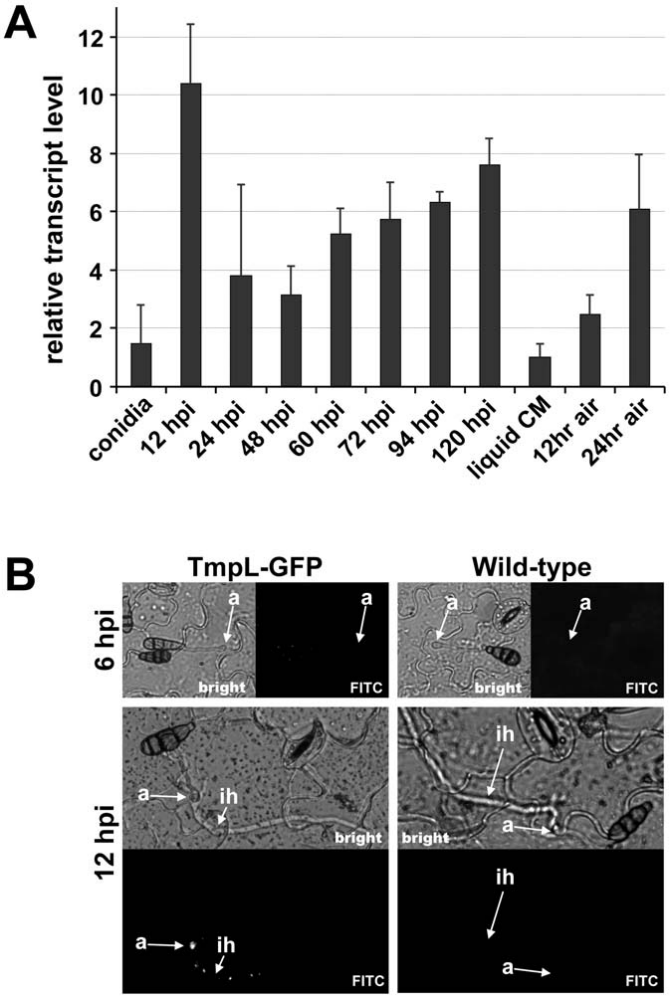
Phase specific expression of *A. brassicicola tmpL*. (**A**) The phase specific expression of *tmpL* was quantified by quantitative real-time (QRT)-PCR after synthesis of cDNA of each developmental RNA including infectious growth, vegetative growth, and conidiation. Relative abundance of *tmpL* transcripts during infectious growth (from ungerminated conidia to *in planta* fungal cells 120 hpi) and conidial development (from 12 hr air-exposed mycelia to 24 hr air-exposed mycelia) was normalized by comparing with vegetative growth in liquid CM (set to transcript level = 1). (**B**) Epifluorescence microscopy of *in planta* GFP expression for the TmpL-GFP strain. The germ tubes and appressoria did not exhibit any GFP signal on the plant surface at 6 hpi. However, a GFP signal was detectable at 12 hpi in a punctate pattern in the appressoria and infection hyphae growing within the plant tissue, consistent with the QRT-PCR results. The wild-type strain was used as a control. Abbreviations: a, appressorium; ih, infection hypha.

To gain further insights into the possible function of TmpL, we next examined *tmpL* mRNA abundance in diverse fungal developmental stages. Relative abundance of *tmpL* mRNA transcripts during vegetative growth, conidiation, and plant colonization were estimated by quantitative real-time polymerase chain reaction (QRT-PCR) (Figure 4A). The abundance of *tmpL* mRNA during vegetative growth in liquid CM was extremely low compared with the internal reference gene, *A. brassicicola* glyceraldehyde 3-phosphate dehydrogenase (*GAPDH*). Interestingly, the mRNA abundance of *tmpL* increased almost six fold at 12 hr post-inoculation (hpi) on plant leaves (i.e., approximately at the time when penetration and infection hyphae develop from appressoria), compared with that of conidia (0 hpi). This result was also supported by *in planta* observation of the TmpL-GFP strain using epifluorescence microscopy (Figure 4B). At 24 and 48 hpi, however, the mRNA abundance was significantly decreased from the 12 hpi level. From 48 hpi, the mRNA abundance gradually increased until 120 hpi. To examine *tmpL* mRNA abundance during conidiation, vegetative mycelia grown in liquid CM were exposed to ambient air to stimulate conidiophore formation and subsequent conidia production. *tmpL* mRNA abundance was gradually elevated up to six-fold during conidiation compared with vegetative growth in liquid CM. Epifluorescence microscopy with the TmpL-GFP strain confirmed the increased expression of *tmpL* in young conidia (Figure 2A) and conidiophores (Figure S3B). Overall, these data indicate that *tmpL* transcript is strongly accumulated during conidiation and during infection *in planta*.

### Targeted mutagenesis of *tmpL* results in abnormal conidiogenesis and accelerated loss of conidial integrity with aging

To further characterize the role of TmpL in fungal development and pathogenesis, a targeted gene replacement strategy was adopted to produce *tmpL* deletion mutants in *A. brassicicola* (Figure S4) and *A. fumigatus* (Figure S5). For the complementation of the *A. brassicicola ΔtmpL* (Ab*ΔtmpL*) strain we introduced both the full-length *tmpL* gene and nourseothricin resistance gene (*NAT*) fragments into the Ab*ΔtmpL* strain. Re-introduction of full-length *tmpL* gene in *A. fumigatus ΔtmpL* (Af*ΔtmpL*) strain was conducted as well by introducing full length *tmpL* with *hph* gene for hygromycin resistance ectopically into the Af*ΔtmpL* strain. The resulting complemented strains were named AbtmpL rec and AftmpL rec for *A. brassicicola* and *A. fumigatus* mutant strains, respectively. All strains were rigorously confirmed with Southern blot and PCR analyses (Figure S4 and S5).

**Figure 5:**
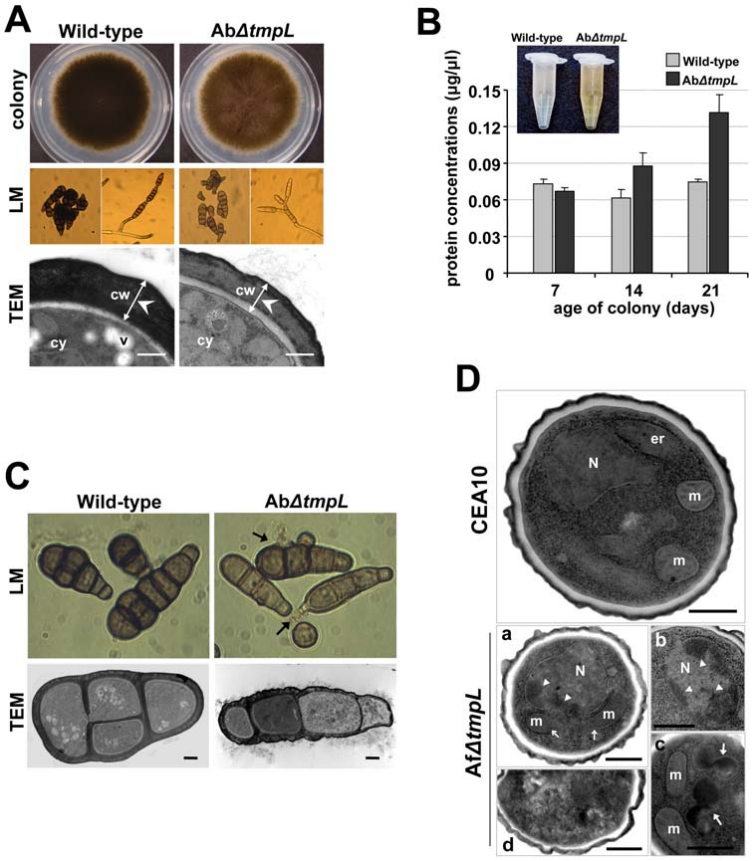
Abnormal conidiogenesis and rapid loss of cell integrity in aged conidia of the *ΔtmpL* mutants. (**A**) Fungal colony grown on solid CM plates (colony). Note that the *A. brassicicola ΔtmpL* mutant colony is a light brown compared with the dark brown color of the wild-type colony. Light micrographs (LM) of less-pigmented conidia and abnormal branching of the conidial chain of the Ab*ΔtmpL* mutants compared to the normal conidiogenesis of the wild-type. Transmission electron micrographs (TEM) depicting the less electron-dense and thinner cell wall of an Ab*ΔtmpL* mutant conidium compared to a wild-type conidium. Bars = 500 nm. Abbreviations: cw, conidial cell wall; cy, conidial cytoplasm; v, vacuole. (**B**) Quantification of protein concentration from conidial suspensions of *A. brassicicola* wild-type and *ΔtmpL* mutant. Note color difference of conidial suspensions between the wild-type and mutants (inset). Values indicate the total quantity of protein released by different-aged fungal cultures from each strain. Average values and SD of three independent quantitations are shown. (**C**) Light and transmission electron micrographs of 21-day-old conidia of *A. brassicicola* wild-type and *ΔtmpL* mutant. Arrows indicate cytoplasmic bleeding due to cell burst of the Ab*ΔtmpL* mutant conidia. Bars = 2 µm. (**D**) Transmission electron micrographs showing sections of 10-day-old conidia of *A. fumigatus* wild-type CEA10 and *ΔtmpL* mutants. Compared to the normal nucleus and subcellular structures of the wild-type conidia, more than half of the Af*ΔtmpL* mutant conidia showed at least one of the apoptotic histological markers: (a) discontinuous or missing mitochondrial outer membrane, (b) chromatin condensation and margination (arrowheads; a and b), (c) accumulation of huge electron dense materials (arrows) in cytoplasm, and (d) conidia with features of necrotic cell death. Bars = 500 nm. Abbreviations: er, endoplasmic reticulum; m, mitochondria; N, nuclei.

Analysis of developmental characteristics, including germination, growth, and conidiation on CM and *in planta*, of *A. brassicicola tmpL* deletion mutants indicated that they were indistinguishable from wild-type and an ectopic mutant A1E1. The mutant strains also showed no defects related to osmotic stress, cell wall perturbation, or responses to antifungal drugs (data not shown). However, it was noted that the Ab*ΔtmpL* strains displayed less pigmentation in culture (Figure 5A). Light microscopy showed that the conidia of the mutants were less pigmented and were narrower than the wild-type. Few multicellular conidia with longitudinal septa were detected among the mutants, which may explain the larger minor axis in wild-type conidia. In addition, increased conidial chain branching was observed in Ab*ΔtmpL* strains compared with the wild-type (Figure 5A). Further investigation of the abnormal mutant conidia using TEM revealed that the conidial cell wall was significantly more electron-dense and thicker in the wild-type than the Ab*ΔtmpL* strain (wild-type, 746±116 nm, n = 53; Ab*ΔtmpL*, 504±83 nm, n = 64; p<0.01). The reconstituted strain AbtmpL rec showed the rescue of the less pigmented conidia and abnormal conidiogenesis seen in the Ab*ΔtmpL* strains (data not shown).

Another interesting difference between *A. brassicicola* wild-type and *ΔtmpL* strains was noticed in older fungal colonies. The conidial suspension of a 21-day-old Ab*ΔtmpL* strain appeared more yellow in color than a comparable wild-type suspension (Figure 5B). We analyzed the conidial suspensions to obtain a secondary metabolite profile using high performance liquid chromatography but the profiles were comparable (data not shown). A protein quantification assay, however, detected large differences in the amount of protein. The 21-day-old Ab*ΔtmpL* strain released more cytoplasm than the wild-type as judged by the amount of total protein quantified in the conidial suspensions (Figure 5B). This result was further supported by our finding that the 21-day-old Ab*ΔtmpL* conidia showed frequent cell bursts in water under light microscopy, which resulted in exuding large amounts of cytoplasm (Figure 5C, LM). Ultrastructural analysis revealed more frequent cell necrosis-like phenotypes in cells of the Ab*ΔtmpL* conidia compared with seemingly intact wild-type conidia (Figure 5C, TEM). In order to clarify the TEM observation, we determined the percentage of old conidia that stained positive with annexin V-FITC, a compound that specifically stains apoptotic or dead cells by binding to phosphatidylserine present on the outer leaflet [58],[59]. The annexin V-stained conidia from 21-day-old Ab*ΔtmpL* strain were increased significantly to 30%, whereas the annexin V-positive wild-type conidia had increased less than 10% after 21 days of growth on CM (Figure S6). These phenotypic abnormalities suggest that the membrane protein TmpL is required for proper fungal conidiation and maintenance of fungal cell integrity with aging in *A. brassicicola*.

*A. fumigatus ΔtmpL* strains displayed no noticeable phenotypic change when grown on glucose minimal media (GMM) plates compared with the wild-type strain CEA10. Unlike *A. brassicicola ΔtmpL* strains, *A. fumigatus ΔtmpL* strains displayed normal pigmentation and cell wall thickness in conidia compared with CEA10 (data not shown). However, when we examined aged conidia using TEM, obvious differences were observed in the Af*ΔtmpL* strain conidia (Figure 5D). The 10-day-old *A. fumigatus* wild-type conidia featured cells with normal structure and clearly identifiable organelles, nuclei surrounded by a nuclear membrane, and mitochondria with well-preserved outer and inner membranes (Figure 5D, CEA10). TEM of the reconstituted strain AftmpL rec conidia were comparable to the wild-type conidia (data not shown). However, Af*ΔtmpL* conidia had an abnormal subcellular morphology (Figure 5D, Af*ΔtmpL*). The mitochondria were less well defined and often displayed discontinuous or missing outer membranes (Figure 5D, a). Chromatin condensation and margination was observed in many nuclei (Figure 5D, a and b) and amorphous electron-dense fragments were frequently aggregated in the cytoplasm (Figure 5D, c). Signs of cell death, such as distorted organelles and numerous small vacuoles, were also observed in some conidia (Figure 5D, d). These features appeared frequently, but not all were observed in every cell.

### Deletion of *tmpL* leads to hypersensitivity to oxidative stresses and excess oxidative burst in fungal cells during conidiation and plant penetration

**Figure 6:**
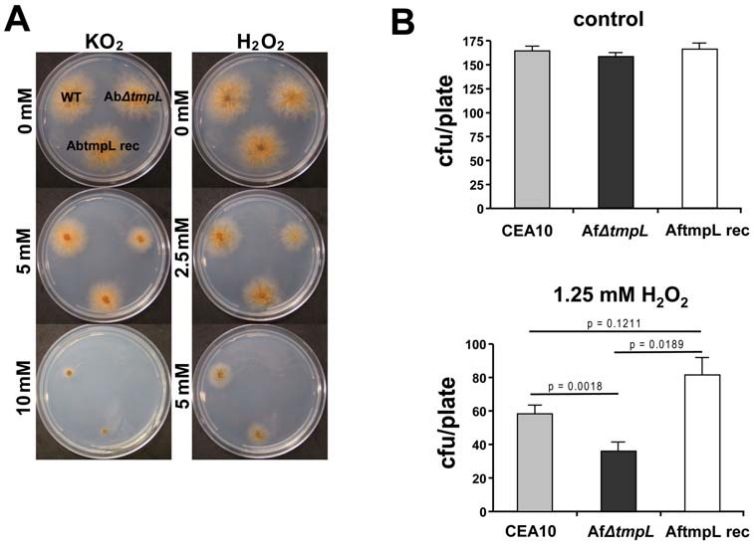
*ΔtmpL* mutants are hypersensitive to extracellular oxidative stress. (**A**) Increased sensitivity of *A. brassicicola ΔtmpL* mutants to oxidative stress generated by KO_2_ or H_2_O_2_ compared with wild-type. Conidial suspensions of *A. brassicicola* wild-type (WT), *ΔtmpL* mutant (Ab*ΔtmpL*), and reconstituted strain (AbtmpL rec) were cultured on minimal agar medium containing different concentrations of KO_2_ or H_2_O_2_ and evaluated 5 days after inoculation. (**B**) Increased sensitivity of *A. fumigatus ΔtmpL* mutant germlings to oxidative stress generated by H_2_O_2_ compared with wild-type. Plates with the germlings of *A. fumigatus* wild-type strain (CEA10), *ΔtmpL* mutant (Af*ΔtmpL*), and reconstituted strain (AftmpL rec) were overlaid with 1.25mM H_2_O_2_ solution, incubated at 37°C for 10 minutes. After washing the plate with sterile distilled water the plates were incubated until colonies were large enough to count. Samples were prepared in triplicate, and error bars on the graph represent SD of two independent experiments.

Given the peroxisomal association of TmpL and the dramatic phenotype during conidiation observed in *ΔtmpL* strains, we suspected a possible involvement of TmpL in oxidative stress responses. To investigate this hypothesis, wild-type and *ΔtmpL* mutants of *A. brassicicola* were examined for sensitivity to two different sources of oxidative stress, the superoxide generator KO_2_ and H_2_O_2_. The Ab*ΔtmpL* strain showed increased sensitivity to oxidative stress compared with the wild-type (Figure 6A). The minimal inhibitory concentration (MIC) of KO_2_ for *A. brassicicola* wild-type was 12.5 mM and for the Ab*ΔtmpL* strain, 7.5 mM; the MIC of H_2_O_2_ for wild-type, 7.5 mM and for Ab*ΔtmpL*, 5 mM. The reconstituted strain AbtmpL rec showed comparable sensitivity to oxidative stress with the wild-type, indicating deletion of *tmpL* caused the hypersensitivity to oxidative stress. In order to investigate the functional conservation of the *A. fumigatus tmpL*, we also examined *A. fumigatus ΔtmpL* strains for sensitivity to oxidative stress. We tested germling sensitivity to H_2_O_2_ for the *A. fumigatus* strains (Figure 6B). The germlings of the Af*ΔtmpL* strain were more sensitive to H_2_O_2_ than the wild-type (p = 0.0018). The reconstituted strain AftmpL rec showed comparable sensitivity to H_2_O_2_ as the wild-type, and a slight, but statistically not significant, increase in tolerance to oxidative stress created by H_2_O_2_ in the germling test (Figure 6B).

**Figure 7:**
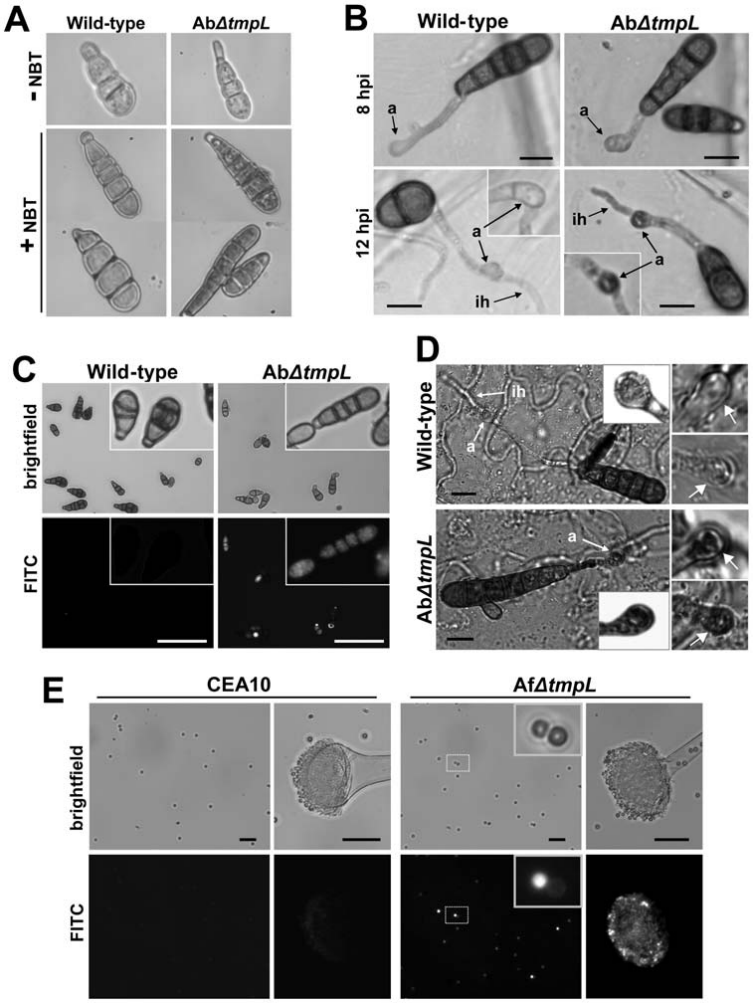
Excess ROS production during conidiation and infection in *ΔtmpL* mutants. (**A**) Accumulation of excess superoxide in the conidia of the *A. brassicicola ΔtmpL* mutants. 7-day-old conidia of *A. brassicicola* wild-type and *ΔtmpL* mutant grown on a nutrient-rich medium were strained with nitroblue tetrazolium (NBT) to detect superoxide. Each micrograph shows the picture of conidium before (−) and after (+) NBT staining. (**B**) Accumulation of excess superoxide in the mature appressoria and emerging infection hyphae of the *A. brassicicola ΔtmpL* mutants. Conidia of *A. brassicicola* wild-type and *ΔtmpL* mutant were inoculated on onion epidermis, incubated at room temperature for 8 and 12 hr, and stained with NBT. Insets show another example of appressorium stained with NBT from each strain. Bars = 10 µm. Abbreviations: a, appressorium; ih, infection hypha. (**C**) Accumulation of excess ROS in the conidia of the *A. brassicicola ΔtmpL* mutants. Conidia released from 7-day-old colonies were stained with H_2_DCFDA and viewed by epifluorescence microscopy. Insets show a magnified view of conidia stained with H_2_DCFDA from each strain. Bars = 50 µm. (**D**) Accumulation of excess H_2_O_2_ in mature appressoria of the *A. brassicicola ΔtmpL* mutants. Conidia of the *A. brassicicola* wild-type and *ΔtmpL* mutant were inoculated on green cabbage cotyledons and incubated at room temperature for 12 hr before being stained with 3,3′-diaminobenzidine tetrahydrochloride (DAB). Insets are the magnified view of each appressorium. Right panels are the pictures of two more appressoria (arrows) stained with DAB from each strain, showing a typical range of staining intensity. Bars = 10 µm. Abbreviations: a, appressorium; ih, infection hypha. (**E**) Accumulation of excess ROS in the conidia of the *A. fumigatus ΔtmpL* mutants. Conidia and conidiophores of the *A. fumigatus* wild-type (CEA10) and *ΔtmpL* mutant were released from 3-day-old colonies and subsequently stained with H_2_DCFDA for 30 min and 1 hr, respectively, and viewed by epifluorescence microscopy. Bars = 20 µm.

Visualization of the accumulation of reactive oxygen species (ROS) was examined to investigate oxygen metabolism during conidiation and plant infection in *A. brassicicola* wild-type and *ΔtmpL* strains. We first investigated the production of ROS by using nitroblue tetrazolium (NBT), which forms a dark-blue water-insoluble formazan precipitate upon reduction by superoxide radicals. Using this technique, it appeared that the Ab*ΔtmpL* strain conidia accumulated higher amounts of superoxide than the wild-type (Figure 7A). Such increased accumulation of superoxide was also detected in the Ab*ΔtmpL* strain inoculated on onion epidermis. Formazan precipitates were typically more intense in the mature appressoria and emerging infection hyphae of the Ab*ΔtmpL* strain, normally after 12 hpi (Figure 7B). However, wild-type appressoria and infection hyphae had less formazan precipitate than the Ab*ΔtmpL* strain.

To investigate production of other ROS in conidia of *A. brassicicola* strains, we used 2′,7′-dichlorodihydrofluorescein diacetate (H_2_DCFDA). This cell-permeable ROS indicator remains nonfluorescent until it is deacetylated by intracellular esterases and oxidized to yield DCF. The H_2_DCF can be oxidized by several ROS generated by intracellular peroxidases, but not directly by H_2_O_2_ [60],[61]. Conidia released from 7-day-old colonies were subject to the H_2_DCFDA staining. More than half of the Ab*ΔtmpL* strain conidia examined were stained by H_2_DCFDA while only few wild-type conidia showed green fluorescence (Figure 7C). Staining with 3,3′-diaminobenzidine tetrahydrochloride (DAB) visualized that mature appressoria of the Ab*ΔtmpL* strain on green cabbage cotyledons also accumulated more H_2_O_2_ than wild-type appressoria at 12 hpi (Figure 7D). Together these data indicate that deletion of *tmpL* in *A. brassicicola* caused an intracellular burst of ROS in conidia and infection structures.

This accumulation of ROS was also visualized in *A. fumigatus* wild-type and the *ΔtmpL* strain conidia using H_2_DCFDA (Figure 7E). H_2_DCFDA staining of conidia from 3-day-old colonies showed a greater intensity of fluorescence in the Af*ΔtmpL* conidia than in the wild-type CEA10 conidia. This brighter fluorescence was detected mainly in the smaller, younger Af*ΔtmpL* conidia (Figure 7E, inset). ROS production appeared to be greater in the conidiophores of Af*ΔtmpL* than wild-type conidiophores, especially in the phialides and not the inflated vesicle of the conidiophore. This indicates that the oxidative burst first takes place mostly within phialides and then young conidia that are formed on the phialides in the absence of the *tmpL* gene in *A. fumigatus*. Taken together, these data indicate that deletion of *tmpL* in *A. fumigatus* resulted in the same phenotype as the *A. brassicicola ΔtmpL* strains: a burst of ROS in conidia and conidiophores.

### Deletion of *A. brassicicola tmpL* causes increased expression of antioxidant genes and nuclear localization of the Yap1 transcription factor during conidiation

**Figure 8:**
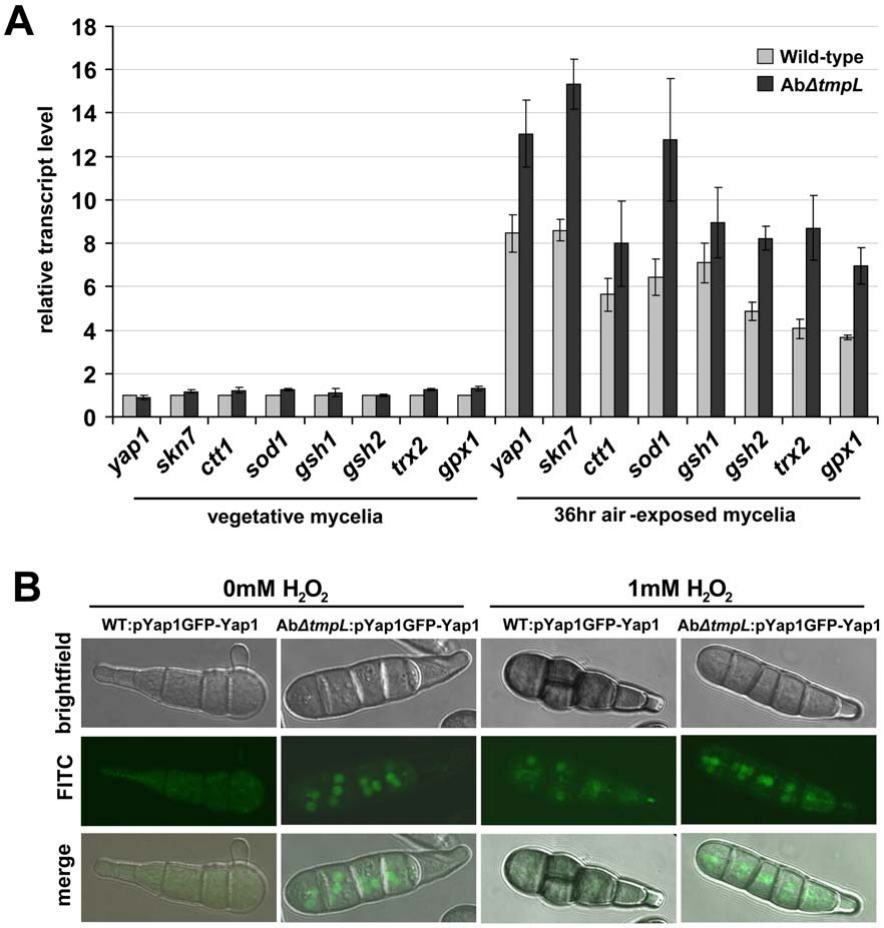
Expression of antioxidant-related genes and nuclear localization of GFP-Yap1 in *A. brassicicola ΔtmpL* mutants. (**A**) Transcript levels of antioxidant-related genes in vegetative mycelia and 36 hr air-exposed mycelia of *A. brassicicola* wild-type and *ΔtmpL* mutant. Relative transcript abundance was determined by comparing each gene transcript level with the transcript level of the same gene in vegetative mycelia of wild-type (set to transcript level = 1). Data are mean±SD of three independent experiments. (**B**) Constitutive nuclear localization of GFP-Yap1 in *A. brassicicola ΔtmpL* mutant conidia. Distribution of GFP-Yap1 in the wild-type (WT:pYap1GFP-Yap1) and the Ab*ΔtmpL* mutant (Ab*ΔtmpL*:pYap1GFP-Yap1) during normal conidiation on CM (0 mM H_2_O_2_) and following treatment of wild-type and Ab*ΔtmpL* mutant with H_2_O_2_ for 1 hr (1 mM H_2_O_2_).

Given the increased ROS accumulation in the absence of TmpL, we next sought to determine whether the ROS scavenging system was impaired in the *ΔtmpL* strains of *A. brassicicola*. We compared the expression of general antioxidant and redox control gene orthologs: *ctt1* (catalase T), *sod1* (Cu,Zn superoxide dismutase), *gsh1* (gamma glutamylcysteine synthetase), *gsh2* (glutathione synthetase), *trx2* (thioredoxin), *gpx1* (glutathione peroxidase 1), and two redox-regulating genes *yap1* and *skn7* in *A. brassicicola* wild-type and *ΔtmpL* strains (Figure 8A). In the wild-type strain, the relative transcript levels of all genes increased up to nine-fold during conidiation (36 hr air-exposed mycelia) compared with the transcript levels in vegetative mycelia. During conidiation all stress-associated genes examined showed up to a two-fold increase in mRNA abundance in the Ab*ΔtmpL* strain compared with the wild-type, while there was a very slight difference observed between the two strains during vegetative growth. Based on the fact that increased ROS levels typically result in higher expression of the enzymes that neutralize them [10],[62], these observations indicate a higher ROS level in the Ab*ΔtmpL* conidia. When combined with excess ROS accumulation observed in Ab*ΔtmpL* conidia (Figure 7), these results also indicate a fundamental inability of the mutant to reduce cellular ROS levels. This may be because it's beyond the cellular capability to neutralize them, even with increased activity of antioxidants. These results also strongly suggest that the Yap1 and Skn7 regulators are not downstream of TmpL activity.

It has been demonstrated in multiple yeast and fungal systems that during oxidative stress, the transcription factor Yap1 facilitates targeted gene expression by migrating into the nucleus from its location in the cytosol [13]. This cellular movement of Yap1 might provide additional information about the state of oxidative stress in the Ab*ΔtmpL* strain. Wild-type and Ab*ΔtmpL* strains were transformed with a GFP-Yap1 construct under the control of the *A. brassicicola yap1* promoter. Cellular localization of the GFP-Yap1 strains was examined by confocal microscopy (Figure 8B). During normal conidiation on solid CM, fluorescence of GFP-Yap1 was distributed evenly throughout the cytoplasm of wild-type conidia (Figure 8B, 0 mM H_2_O_2_). In contrast, the Ab*ΔtmpL*:pYap1-GFP-Yap1 strains showed a focal, condensed GFP signal typical of nuclear localization, suggesting the mutant is in a state of constitutive oxidative stress during conidiation. By constrast, there was cytoplasmic distribution of the GFP signals observed in mycelia of the Ab*ΔtmpL*:pYap1-GFP-Yap1 strains (data not shown). This observation not only indicates excess ROS accumulation only in conidial cells, but also excludes any possible involvement of environmental factors generating ROS in fungal cells, such as UV radiation, temperature shift, mechanical damage, etc [63]. In a parallel experiment, treatment of WT:pYap1-GFP-Yap1 and Ab*ΔtmpL*:pYap1-GFP-Yap1 strains with 1 mM H_2_O_2_ for 1 hr resulted in substantial nuclear localization of GFP-Yap1 in both strains (Figure 8B, 1 mM H_2_O_2_). This indicates that the GFP-Yap1 proteins in both strains are functional. Staining with DAPI confirmed our observations that GFP-Yap1 was indeed localized to the nucleus in these experiments (data not shown).

### TmpL is required for *A. brassicicola* and *A. fumigatus* virulence

**Figure 9:**
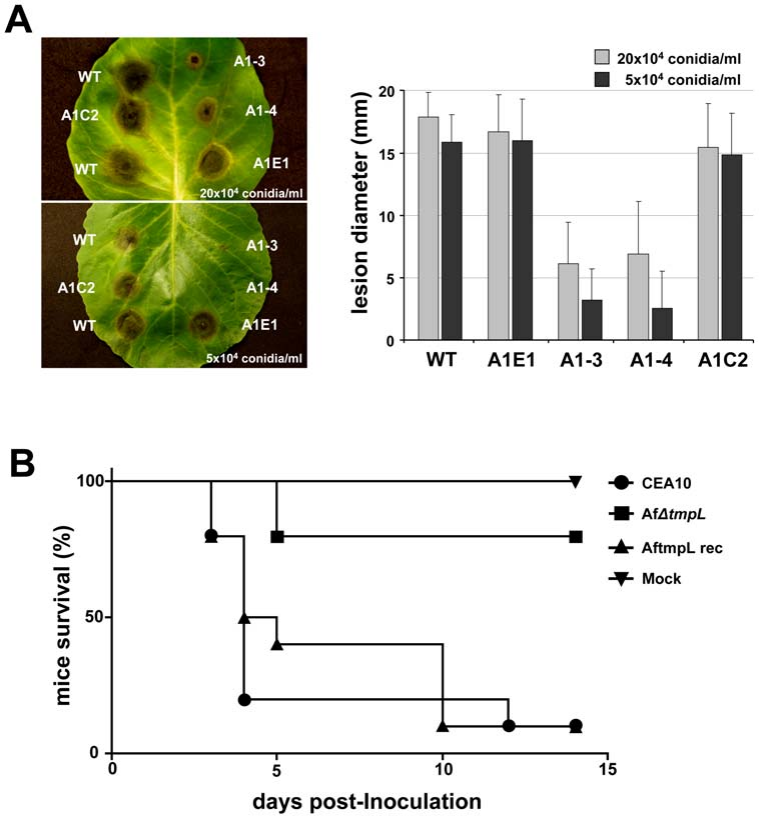
Reduced virulence of *ΔtmpL* mutants. (**A**) Virulence assay on green cabbage leaves using two conidial concentrations, 20×10^4^ and 5×10^4^ conidia ml^−1^ of *A. brassicicola* wild-type (WT), ectopic (A1E1), two *ΔtmpL* mutants (A1–3 and A1–4), and a reconstituted strain (A1C2). Five days after inoculation (graph), disease severity was calculated based on the lesion diameter. Columns and error bars represent average and SD, respectively, of five independent experiments. (**B**) Virulence assay on mice mock inoculated or inoculated intranasally with 10^6^ conidia/ 25 µl of *A. fumigatus* wild-type (CEA10), *ΔtmpL* mutant (Af*ΔtmpL*), and reconstituted strain (AftmpL rec). P value for comparison between Af*ΔtmpL* mutant and wild-type CEA10, *P* = 0.0006. Af*ΔtmpL* is significantly less virulent than the wild-type CEA10 and the reconstituted strain AftmpL rec.

Given the above phenotypes of the *ΔtmpL* strains, we hypothesized that TmpL may play a key role in fungal virulence. To investigate the role of TmpL in *A. brassicicola* virulence, susceptible green cabbage (*Brassica oleracea*) were inoculated with two different concentrations of young, 7 day old conidia (2×10^5^ and 5×10^4^ conidia ml^−1^) (Figure 9A). Plants inoculated with either wild-type or ectopic mutant (A1E1) developed extensive, typical black spots on leaves at both concentrations of conidia tested. However, the black necrotic spots resulting from inoculation with Ab*ΔtmpL* strains (A1–3 and A1–4) at both conidial concentrations was significantly smaller than those produced by the wild-type or ectopic mutant inoculations (p<0.01). The reconstituted strain AbtmpL rec (A1C2) was found to be just as virulent as the wild-type at both concentrations of conidia. The average reduction in disease severity caused by the mutants compared with the wild-type was more than 62% and 80% when using the higher and lower conidial concentrations, respectively. Similar results were obtained in virulence assays with *Arabidopsis*.

We next asked the question whether *tmpL* is also involved in fungal virulence in the human fungal pathogen *Aspergillus fumigatus*. Deletion of *tmpL* in *A. fumigatus* led to a statistically significant reduction (p<0.01) in virulence in a chemotherapeutic murine model of invasive pulmonary aspergillosis (Figure 9B). Mice infected with the Af*ΔtmpL* strain did not display normal symptoms associated with invasive aspergillosis (IA) in contrast to wild-type and reconstituted strain infected mice which displayed well described symptoms of IA including ruffled fur, hunched posture, weight loss, and increased respiration. Consequently, like the *ΔtmpL* mutant in *A. brassicicola* that has reduced virulence on plants, TmpL is also required for fungal virulence in mammalian hosts.

### *A. brassicicola ΔtmpL* strains fail to penetrate plant tissue and induce active callose deposition *in planta*

**Figure 10:**
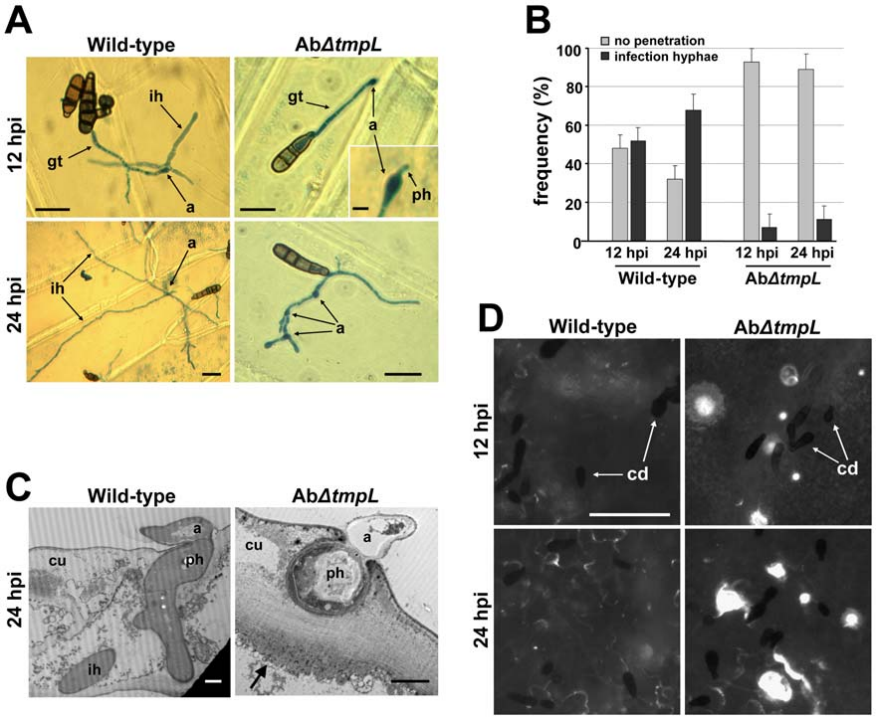
Appressoria and infection hyphae formation, ultrastructure, and callose detection assays of *A. brassicicola ΔtmpL* mutant infection. (**A**) Light micrographs of onion epidermis inoculated with *A. brassiciola* wild-type and *ΔtmpL* mutants at 12 and 24 hpi. Note that penetration hyphae (inset) were frequently visible under the mutant appressoria, but no further development was observed in the Ab*ΔtmpL* mutant infection. Bars = 20 µm, except for the inset where it denotes 5 µm. Abbreviations: a, appressorium; gt, germ tube; ih, infection hypha; ph, penetration hypha. (**B**) Frequency of infection hyphae formation beneath appressoria of the *A. brassicicola* wild-type and *ΔtmpL* mutants on onion epidermis. Columns and error bars represent average and SD, respectively, of four independent experiments. (**C**) Transmission electron micrographs showing transverse sections of green cabbage leaves inoculated with *A. brassicicola* wild-type and *ΔtmpL* mutants. Leaves were collected at 24 hpi and prepared for transmission electron microscopy. Arrow indicates papilla formation (callose deposition) around a fungal penetration hypha. Bars = 2 µm. Abbreviations: a, appressorium; cu, cuticle layer; ih, secondary infection hypha; ph, penetration hypha. (**D**) Callose deposition on green cabbage cotyledons inoculated with *A. brassicicola* wild-type and *ΔtmpL* mutants. White spots indicate callose deposition of the inoculation sites stained by aniline blue and viewed by epifluorescence microscopy. The tiny black spots dispersed on plant surface are fungal conidia (cd). Bars = 50 µm.

To understand the reasons for the reduced virulence of *A. brassicicola ΔtmpL* strains on green cabbage, we performed microscopic analyses of the infection processes. Examination of green cabbage cotyledons using light microscopy at 12 hpi revealed that the mutants formed appressoria on the plant surface similar to those formed by wild-type (Figure S7A). Intracellular infection hyphae formed directly under the appressoria of the Ab*ΔtmpL* strain, however, rarely developed inside of plant epidermal cells, while development of infection hyphae from wild-type appressoria was consistently observed. An onion epidermis assay also showed similar results as the cotyledon assay (Figure 10A). Only 7% of Ab*ΔtmpL* appressoria produced visible intracellular infection hyphae at 12 hpi (Figure 10B), but initial penetration hyphae from most individual appressoria were frequently visible (Figure 10A, inset). At 24 hpi, ∼11% of the Ab*ΔtmpL* appressoria developed intracellular infection hyphae. The remaining Ab*ΔtmpL* appressoria did not develop infection hyphae, but in some cases, produced one or several germ tubes that formed additional appressoria (Figure 10A, 24 hpi). In contrast, more than half of the wild-type appressoria successfully produced intracellular infection hyphae at 12 hpi (Figure 10B), which usually penetrated cross-walls and spread within 24 hr (Figure 10A, 24 hpi).

To characterize the host-pathogen interface, inoculated green cabbage leaves were examined by light and electron microscopy. In vertical leaf sections inoculated with the compatible wild-type, fungal appressoria successfully penetrated, formed intracellular infection hyphae, and killed most plant tissue below the infection sites within 24 hr (Figure S7B and 10C). In contrast, leaf sections inoculated with the less virulent Ab*ΔtmpL* strain appeared undamaged, though it was noted that necrosis similar to a hypersensitive response or papillae formation (callose deposition) developed below the infection site (Figure S7B). Transmission electron microscopy revealed penetration hyphae and appressoria of the Ab*ΔtmpL* strain showing typical cell death-like phenotypes (cytoplasmic fragmentation, enlarged vacuoles, and distorted organelles) and the penetration hyphae were completely arrested by papillae formation in plant epidermal cells (Figure 10C). Callose deposition was also detected by cytological staining using aniline blue (Figure 10D). The wild-type induced small, scattered deposits in close proximity to the sites of penetration and tissue necrosis was extensive. In contrast, callose deposits observed following Ab*ΔtmpL* inoculation were much more pronounced and often localized at the site of penetration.

In order to investigate whether the Ab*ΔtmpL* strains can colonize the host plant when the first physical barrier, the plant cell wall, is removed, wounded leaf assays were performed (Figure S7C). Symptoms produced by inoculation of the wild-type on wounded tissue were more severe than on intact (non-wounded) tissue. The Ab*ΔtmpL* strain formed larger lesions on wounded leaves than on intact leaves, but were still smaller than wild-type lesions on wounded leaves.

Together, these results indicate that *A. brassicicola ΔtmpL* strains have defects in pathogenicity associated primarily with very early stages of plant infection, resulting in the failure of appressoria penetrating into epidermal cells and an induction of callose deposition.

### *A. fumigatus ΔtmpL* strains exhibit reduced colonization in inoculated mice

**Figure 11:**
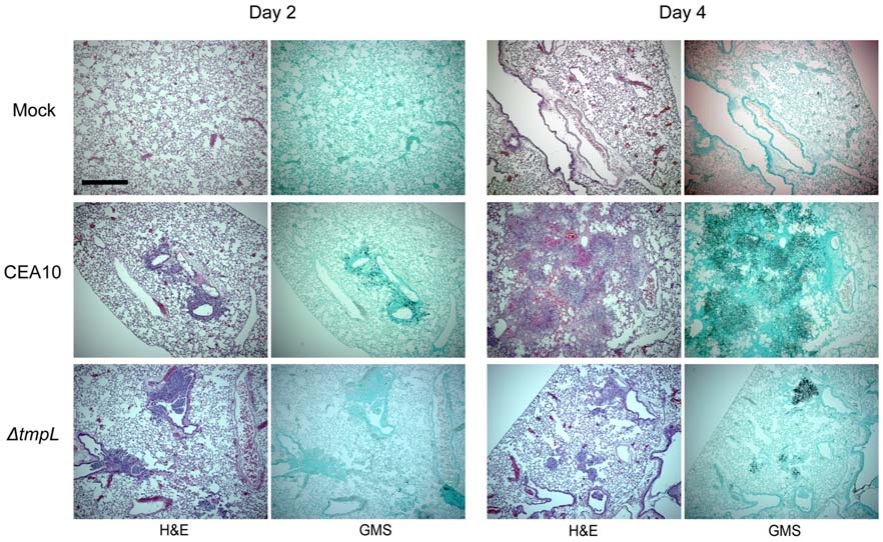
Representative histopathology of CD-1 mouse model infected with *A. fumigatus* wild-type and *ΔtmpL* mutants. Mock = 0.01% Tween inoculated, CEA10 = WT, *ΔtmpL* = *A. fumigatus ΔtmpL* mutant. Mice were inoculated with 1×10^5^ conidia intranasally, euthanized on days +2 and +4 after inoculation, lungs removed, fixed in formaldehyde, and stained with hematoxylin and eosin (H&E) or Gommori's methenamine silver (GMS) stain. On day +2, Af*ΔtmpL* mice generally displayed less necrotic lesions and less fungal burden as the wild-type. Significant inflammation, necrosis, and an influx of immune effector cells (primarily neutrophils) were observed on day +4 in all infected animals but not the mock control. However, lesions are more localized and not as extensive and we clearly observed more open alveoli in mice infected with the Af*ΔtmpL* strain. Interestingly, GMS staining revealed that fungal growth is less extensive in the Af*ΔtmpL* strain as well. Bar = 500 µm at 40× magnification.

To further understand the potential mechanism behind the virulence defect of the *A. fumigatus ΔtmpL* mutant, we examined lung histopathology from mice on days +2 and +4 of the infection. On day 2, Af*ΔtmpL* mice generally displayed less necrotic lesions and less fungal burden as observed by H&E and GMS stains (Figure 11). However, the differences with regard to inflammation were subtle between wild-type and mutant infected animals and it is clear that both fungal strains were able to germinate and colonize the lung tissue (Figure 11). QRT-PCR analysis of fungal burden based on amplification of fungal 18S rRNA revealed an approximate 10 fold decrease in fungal burden in mice infected with the Af*ΔtmpL* mutant (data not shown). However, by day 4, both wild-type and Af*ΔtmpL* mutant mice displayed significant histopathological findings associated with *Aspergillus* infections including the development of granulomatous like lesions, massive influx of inflammatory cells (primarily neutrophils) to sites of infection, subsequent peribronchiolar and alveolar inflammation, and substantial fungal growth in silver stained tissue (Figure 11). In general, the inflammation and necrosis observed was much more significant in wild-type infected animals than Af*ΔtmpL* infected animals (Figure 11). However, it was clear that the Af*ΔtmpL* mutant was still persistent and causing pathology at this time point. These results partially mimic findings with regard to the virulence of the *A. brassicicola ΔtmpL* mutant during infection of wounded plants that displayed a slower colonization and disease progression than the wild-type strain. With regard to these animal experiments, it is unclear if the slower colonization of the mouse lung tissue by the Af*ΔtmpL* strain observed on day 2 and day 4 of the infection is due to lack of growth by the fungus in the *in vivo* environment, or improved clearance by the host immune response. Additional studies are ongoing to further characterize the mechanism behind the virulence defect of the Af*ΔtmpL* mutant strain.

### Overexpression of *yap1* in *A. brassicicola ΔtmpL* background leads to partial complementation of abnormal conidiation, oxidative stress tolerance, and reduced virulence

**Figure 12:**
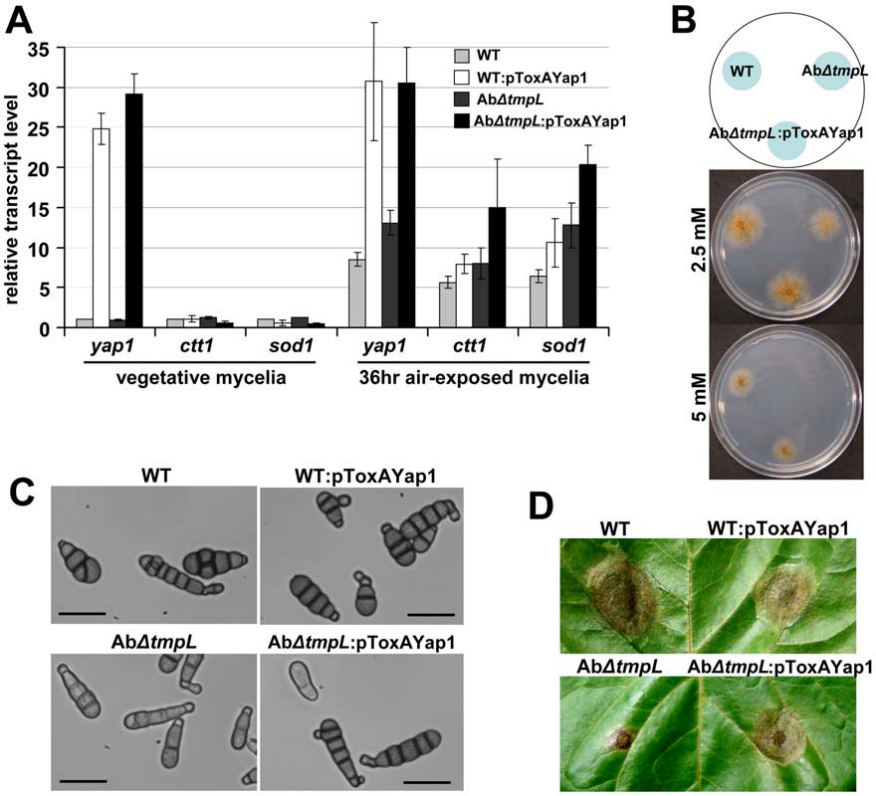
Restoration of the abnormal phenotypes and reduced virulence of *A. brassicicola ΔtmpL* mutants by redox regulator *yap1* overexpression. (**A**) Transcript levels of *yap1*, *sod1*, and *ctt1* in vegetative mycelia and 36 hr air-exposed mycelia of *A. brassicicola* wild-type (WT), *yap1* overexpression mutant on wild-type background (WT:pToxAYap1), *ΔtmpL* mutant (Ab*ΔtmpL*), and *yap1* overexpression mutant on *ΔtmpL* mutant background (Ab*ΔtmpL*:pToxAYap1). Relative transcript abundance was determined by comparing each gene transcript level with the transcript level of the same gene in vegetative mycelia of wild-type (set to transcript level = 1). Data are mean±SD of two independent experiments. (**B**) Hypersensitivity to extracellular oxidative stress generated by H_2_O_2_ was recovered by *yap1* overexpression in Ab*ΔtmpL* mutants. Conidial suspensions of WT, Ab*ΔtmpL*, and Ab*ΔtmpL*:pToxAYap1 strain were cultured on minimal agar medium containing different concentrations of H_2_O_2_ and evaluated 5 days after inoculation. (**C**) Light micrographs showing restoration of the abnormal conidiogenesis of Ab*ΔtmpL* mutants in Ab*ΔtmpL*:pToxAYap1 overexpression strain. Conidia released from 7-day-old colonies of WT, WT:pToxAYap1, Ab*ΔtmpL*, and Ab*ΔtmpL*:pToxAYap1 strain were observed with a light microscope. Note that *yap1* overexpression in the wild-type has no significant effect on the conidiogenesis, resulting in similar conidia production. Bars = 20 µm. (**D**) Partial restoration of the reduced virulence of Ab*ΔtmpL* mutants in Ab*ΔtmpL*:pToxAYap1 overexpression strain. Note that *yap1* overexpression in the wild-type negatively affected *A. brassicicola* pathogenicity, resulting in smaller lesion development compared to the wild-type. Pictures were taken 5 days after inoculation.

Given the excess oxidative burst phenotypes of the *ΔtmpL* strains, we hypothesized that overexpression of *yap1* may rescue the *ΔtmpL* mutant phenotypes. To determine whether overexpression of the Yap1 transcriptional regulator can enhance the cellular scavenging ability of fungal cells and consequently restore the abnormal phenotype and reduced virulence in *ΔtmpL* strains, we generated a *ToxA* promoter-driven *yap1* overexpression cassette using fusion PCR methods. Subsequently, we introduced the overexpression cassette into both *A. brassicicola* wild-type and *ΔtmpL* backgrounds and examined its effect on each strain. As shown in Figure 12A, the mRNA abundance of *yap1* significantly increased at least 25-fold compared with each recipient strain: wild-type and Ab*ΔtmpL*, indicating that *yap1* overexpression cassettes were successfully integrated in the genome and expressed under the control of the *ToxA* promoter. To evaluate whether Yap1 overproduction affected the induction of the antioxidant defense system, we monitored the transcriptional activation of *ctt1* and *sod1* orthologs as representative downstream genes regulated by Yap1. During vegetative growth, there was no induction of the *ctt1* and *sod1* transcripts. During conidiation in 36 hr air-exposed mycelia, however, the *yap1* overexpression mutant in the Ab*ΔtmpL* background (Ab*ΔtmpL*:pToxA-Yap1) showed significantly increased expression (almost two-fold) of antioxidant genes. Yet, *yap1* overexpression in the wild-type (WT:pToxA-Yap1) resulted only in a slight increase of these antioxidant genes, possibly because of the mechanism of Yap1 activation; Yap1 is post-translationally activated only in the presence of cellular ROS [13],[64].

Overexpression of *yap1* restored oxidative stress tolerance of the Ab*ΔtmpL* strain, resulting in comparable sensitivity to H_2_O_2_ as the wild-type (Figure 12B). Furthermore, the Ab*ΔtmpL*:pToxA-Yap1 strain produced wild-type-like conidia (Figure 12C), indicating that *yap1* overexpression complemented, at least to a substantial degree, the *ΔtmpL* phenotypes. There was no distinguishable phenotypic difference between the WT:pToxA-Yap1 strain and the wild-type recipient strain. In addition to the conidial phenotype, green cabbage infection assays showed that the Ab*ΔtmpL*:pToxA-Yap1 strain partially restored its virulence compared with the Ab*ΔtmpL* recipient strain, but was still not comparable to the wild-type (Ab*ΔtmpL*, 4.1±2.83 nm, n = 26; Ab*ΔtmpL*:pToxA-Yap1, 12.9±4.52 mm, n = 26; p<0.01) (Figure 12D). Interestingly *yap1* overexpression in the wild-type caused slightly decreased lesion size compared with its wild-type recipient strain (wild-type, 17.2±2.5 mm, n = 22; WT:pToxA-Yap1, 15.7±3.8 mm, n = 22; p<0.05), indicating that excess antioxidant activity resulting from *yap1* overexpression did indeed negatively affect the pathogenesis of the *A. brassicicola* wild-type. Overall, *yap1* overexpression in the Ab*ΔtmpL* strain strongly suggested that the phenotypic defects and reduced virulence were attributable to failure in the regulation of intracellular ROS levels, particularly in conidia and infection-related structures during the conidiation process and during plant infection, respectively. However, the residual virulence defect in the presence of *yap1* overexpression may suggest additional roles of *tmpL* in fungal virulence.

## Discussion

Mechanisms for adapting to stress either from intracellular or extracellular sources are among the most relevant and timely topics in fungal biology. During normal developmental processes, a fungal organism encounters various stresses from toxic by-products of its metabolism or oxidative stress generated mainly through aerobic respiration [33],[65]. The cellular environment within a host, whether plant or animal, also represents a major source of stress to an invading fungal pathogen [26],[66],[67]. In order to evade or circumvent stress, the fungus must possess special adaptation mechanisms. In this study we provide the first evidence that a novel, pathogenicity-related gene from a plant and animal fungal pathogen, *tmpL*, is critical for proper conidiogenesis and infection of healthy host tissues. Furthermore, *tmpL* appears to be associated with a filamentous fungi-specific stress defense system that particularly responds to oxidative stress.

TmpL is a novel hybrid protein consisting of an AMP-binding domain, six putative transmembrane domains, and a FAD/NAD(P)-binding domain. Based on our phylogenetic analysis, TmpL and its putative orthologs are present only in filamentous fungi (Figure S1) and not highly related to proteins with known functions. Although portions of the predicted TmpL amino acid sequence showed high similarity to putative NPS protein sequences in the GenBank NR database, its sequence lacked thiolation and condensation domains necessary to create a minimal module in typical NPS proteins. The AMP-binding domain is very similar to an adenylation domain. The latter is most often associated with modular NPS enzymes, where it activates amino acids prior to their incorporation into nonribosomal peptides (NRP) [68]. Interestingly, all fungi that contained a TmpL homolog also contain numerous *NPS* genes. Though the exact function of TmpL remains to be determined, it may modify or activate specific amino acids associated with certain nonribosomal peptides acting as a signal molecule for oxidative stress responses in filamentous fungi. It is also proposed that based on the similarity of the C-terminal sequences of TmpL to a previously identified, although smaller, plasma membrane flavoprotein in *A. nidulans*, TmpA, TmpL might be involved in production of a regulatory signal, which eventually leads to fungal differentiation. As predicted in TmpA [36], we suspected that the C-terminal region of TmpL had enzymatic activity. Bioinformatic analysis also showed TmpL and its orthologs contain proposed sites for FAD and NAD(P)-binding, based on protein modeling and the existence of two important consensus sequences, suggesting that the protein is specifically reduced by NAD(P)H with a reduction potential. Indeed in our study, a partial recombinant protein of TmpL, which includes FAD/NAD(P)-binding domain, supports this hypothesis by showing that the partial protein is capable of binding flavin. In addition, NCBI conserved domain BLAST searches identified a ferric reductase (FRE) domain with low similarity (*E*-value 0.004) in the FAD/NAD(P)-binding domain of the TmpL protein, suggesting that TmpL might be distantly related to the FRE group of proteins. Indeed several FRE proteins are known to be involved in the response to oxidative stress in various organisms [69],[70], as part of a system that activates a number of different enzymes involved in redox control. When considered together, it is likely that TmpL uses electrons from NAD(P)H, transferred via FAD, to activate or modify unknown substrates or possibly downstream proteins in a redox-related signal transduction pathway.

Our localization assays indicated that TmpL is associated with the Woronin body (WB) in filamentous fungi. WBs are known to plug septal pores in response to fungal cell injury, preventing excess cytoplasmic leaking [57],[71]. Early TEM studies indicated a peroxisomal origin for WBs [72]. More recently, genetics and cell biology research confirmed that the WB is first assembled in large peroxisomes [54],[55]. Our confocal microscopy analysis showing a sequential association between TmpL and peroxisomes suggests that TmpL is first targeted into peroxisomes by an unknown peroxisomal targeting signal and then goes through WB biogenesis, eventually becoming part of a mature WB. However, WB in *A. brassicicola* conidia appeared to be divided into two groups based on their location and the localization of TmpL. It is generally accepted that depending on the organism, cell type, and metabolic requirements, distinct sets of proteins could be housed within certain multipurpose organelles or microbodies [53],[73]. Confocal analyses with TmpL-GFP and DsRed-AbHex1 double-labeled strain and TEM analysis of *A. brassicicola* conidia showing existence of one or two WB located in the cytoplasm near the cell cortex support this hypothesis. In addition, cytoplasmic redistribution of the TmpL-GFP fluorescence in a *Δpex14* strain indirectly, albeit strongly, supports the idea that TmpL is associated with a specific WB where AbHex1 is localized. Several reports on WB from other fungi have established the presence of WB in non-septal regions, such as the tips of the germlings and secondary infectious hyphae, or at the cell periphery [74],[75],[76]. These WB showed no association with the hyphal septum, suggesting other possible functions than plugging septal pores in response to cell injury. For example, loss of WB in *Magnaporthe grisea Δhex1* strains led to increased cell death in response to nitrogen starvation. This suggests that WB may function in response to environmental stress [76]. PRO40, associated with WB in *Sordaria macrospora*, was pivotal in triggering the developmental switch from protoperithecia to perithecia [52]. Together, these findings indicate other possible functions of the WB associated with development or the multicellular growth characteristic of filamentous fungi. On the other hand, it is also true that very little is known about the WB function in other fungal structures such as conidia and specialized infection structures. Although we cannot rule out the possibility that DsRed-AbHex1 was targeted incorrectly to the peroxisome-like organelles where TmpL-GFP was localized because of its ectopic expression, it is more likely that these observations reflect the existence of a specific WB which is associated with TmpL. To confirm the association between TmpL and WB in the future, more detailed biochemical analyses are needed. These include either immunodetection assays using TmpL- and Hex1-specific antibodies following differential and density gradient centrifugation, or immunofluorescence microscopy.

It has been well documented that regulation of ROS level is important during fungal development [21],[63]. In this study, we also highlighted the significance of intracellular ROS concentration in relation to fungal development. Given the observations that *tmpL* was highly expressed during conidiation and the loss-of-function mutation resulted in abnormal conidiogenesis and excess ROS accumulation in conidia, we can speculate that TmpL is involved in important mechanisms for balancing ROS level during conidiation. Deletion of a catalase gene (*CATB*) in *M. grisea* caused similar phenotypic changes as was observed in the *ΔtmpL* strains, such as less pigmentation, fragile conidia, and reduced virulence [77], indicating a possible common effect of excess intracellular ROS in filamentous fungi. In many fungi, inhibition of ROS generation or excess intracellular ROS levels affected various fungal developmental processes [6],[35],[63],[78]. Even a fungus-plant mutualistic symbiosis requires a sophisticated regulation of the ROS production [79],[80]. Consistent with the involvement of ROS in cell-wall biosynthesis [81], it seems probable that the excess ROS levels in *ΔtmpL* strains resulted in lighter pigmentation in the *A. brassicicola* conidia. Several studies also reported that accumulation of ROS within the cytoplasm played a central role in apoptosis-like cell death [82],[83], as shown in our observations of apoptosis-like cell death phenomena in aged conidia of both *A. brassicicola* and *A. fumigatus ΔtmpL* strains.

Increased expression of antioxidant genes in *A. brassicicola ΔtmpL* strains is another indicator of increased ROS levels in the cell. Indeed, several reports in different microorganisms have shown a correlation between the up-regulation of specific antioxidant enzymes and increased cellular ROS levels [21],[84],[85], suggesting that increased ROS levels result in higher expression of the enzymes that neutralize them. On the other hand, it could be questioned why the increased antioxidant expression in the *ΔtmpL* strains did not result in reducing cellular ROS levels in the mutant cells. The possible reason for that would be excess ROS levels in the *ΔtmpL* strains were far beyond the cellular capability (or threshold) to neutralize them. Our results from experiments of *yap1* overexpression in *ΔtmpL* mutant background provide major evidence for this hypothesis. Upon oxidative stress, Yap1 is involved in activating genes involved in a cellular antioxidant system, such as *GSH1* (γ-glutamylcysteine synthetase), *TRX2* (thioredoxin), *GLR1* (glutathione reductase), and *TRR1* (thioredoxin reductase) [86]. Therefore we can speculate that Yap1 overproduction led to the increase of the cellular antioxidant defense capability in the *ΔtmpL* strain that produces excess intracellular ROS in conidia. Indeed, *yap1* overexpression suppressed most of the phenotypic defects shown in the *ΔtmpL* strain, indicating excess intracellular ROS was most likely the primary reason for the phenotypic changes observed in the *ΔtmpL* mutants. Interestingly *yap1* overexpression in the wild-type strain did not affect the expression levels of downstream antioxidant genes *ctt1* and *sod1*, consistent with the post-translational activation model of the Yap1 protein by intracellular ROS. When considered together, these results demonstrate that TmpL may be associated with a filamentous fungi-specific oxidative stress defense system. However, we cannot rule out another possibility that TmpL is involved in cellular ROS production. As a consequence of the loss of TmpL-operated ROS production, an additional means of ROS generation may be up-regulated during conidiation, resulting in excess production of ROS. Indeed *M. grisea Δnox1Δnox2* mutant displayed increased ROS generation during hyphal growth compared with wild-type strain [6], indicating that there is an alternative ROS source that is activated upon loss of the Nox enzymes. Similarly, in *Podospora anserina* inactivation of *panox1* led to an enhanced ROS production in mycelia [87]. However there was no difference observed in the expression levels of *A. brassicicola nox* homologs, *AbnoxA* and *AbnoxB* between wild-type and *ΔtmpL* stains during conidiation process (data not shown), suggesting the NADPH oxidase-mediated ROS production is not the cause of excess oxidative stress in the *ΔtmpL* stains.

A major question from our work is the role of TmpL in fungal virulence. We observed that loss of TmpL function resulted in avirulence in both plant and animal fungal pathogens. With regard to plant pathogenesis, *A. brassicicola ΔtmpL* conidia successfully germinated and formed normal appearing appressoria on plant surfaces at similar rates as wild-type. Thus, a defect in germination or appressoria development cannot explain the mutant phenotype during plant pathogenesis. However, only 7% of the total appressoria were capable of penetrating the host and growth was rapidly arrested in the epidermal cells. Additionally, the mutant appressoria and penetration hyphae observed by TEM showed a cell-death-like phenotype that we speculate may be due to excess oxidative stress, as indicated by NBT and DAB staining. To understand whether the infection failure in *ΔtmpL* strains was related to the excess buildup of ROS therein, we tried to reduce the levels of ROS during *in planta* appressoria development and penetration using a NADPH oxidase inhibitor diphenylene iodonium or antioxidant ascorbic acid. However, none of the treatments were successful in restoring the infection failure of the *ΔtmpL* strains. Even the infection of wild-type strains treated with these agents was seriously suppressed and resulted in tiny lesions on host leaves (data not shown). The latter result seems to be explained by the same reasoning with the observation that *yap1* overexpression in the wild-type strain caused reduced lesion size compared with its wild-type recipient strain. All of these results suggest that an excess reduction in intracellular but not extracellular oxidative stress also leads to a significant suppression of fungal infection. In other words, a sophisticated balancing of ROS levels is critical in fungal pathogenesis of plants. As an alternative method of reducing excess ROS in appressoria and/or penetration hyphae of the *ΔtmpL* mutants, we chose to manipulate the existing antioxidant system present in filamentous fungi by overexpressing *yap1*. NBT staining showed less superoxide accumulation in the appressoria of the Ab*ΔtmpL*:pToxA-Yap1 overexpression strain compared with the Ab*ΔtmpL* recipient strain (data not shown). Although the overexpression strain exhibited significantly restored virulence, it still was not comparable to the wild-type. Thus, our *yap1* overexpression analyses clearly demonstrated that the infection failure in *ΔtmpL* strains was related to the intracellular accumulation of excess ROS in fungal infection structures.

Regulation of ROS level during pathogenesis has been a critical factor that governs success or failure of the infection process. For example, *M. grisea* showed considerable amount of oxidative burst in appressoria during its pathogenesis, and inhibition of the ROS production by some inhibitors resulted in abnormal appressoria and further failure of plant infection [6]. Deletion of the Yap1 oxidative stress response protein in *Ustilago maydis* caused avirulence on corn, resulting from an excess oxidative stress on infection structures [19]. In addition, numerous fungal pathogens of animals have been reported to possess a defined genetic program to respond to oxidative killing by the host [10],[88],[89],[90]. However, *yap1* deletion mutants in the human fungal pathogen *A. fumigatus* are still virulent in chemotherapeutic models of invasive aspergillosis [91]. This observation, coupled with the lack of full virulence restoration in the *A. brassicicola ΔtmpL* mutant strains overexpressing *yap1* may suggest that the virulence defect of *tmpL* deficient strains is due to additional unknown causes. Indeed, in our studies the virulence of the *A. fumigatus ΔtmpL* mutant was also attenuated in gp91^phox−/−^ mice, which are deficient in generating a respiratory burst and highly susceptible to *A. fumigatus* infection (data not shown). Collectively, these studies and our observations suggest that production and accumulation of excess intracellular ROS, and not increased sensitivity to extracellular ROS, in both *ΔtmpL* mutants of plant and animal pathogenic fungi is the primary cause for reduced virulence. Thus increased sensitivity to and detoxification of host derived, extracellular ROS, is most likely not the reason for the avirulence observed in *ΔtmpL* mutants in both pathosystems. Recent discoveries of functional ROS-generating enzymes within filamentous fungi have elucidated some possible roles of the fungus-derived ROS in pathogenic species [6],[92]. Fungal contributions to ROS production have been obtained from fungi showing such activity without any contact of host cells. For example, spores of *M. grisea* germinating in water generated H_2_O_2_, O_2_^−^, and OH**^+^** extracellularly [93] and ROS production was associated with the development of infection structures on glass coverslips [6]. Previous studies have also speculated the possible involvement of fungus-derived ROS production in the rapid growth and spread of the pathogens inside their hosts [94],[95]. Together with the excess ROS accumulation in the *ΔtmpL* conidia it is more plausible to speculate that the failure of regulating intracellularly produced ROS caused the penetration failure of the *ΔtmpL* strains, and thus the reduced virulence. While *A. fumigatus* is not known to produce penetration structures like appressoria to invade mammalian hosts, it may be possible that failure to handle fungal ROS accumulation during the initial stages of host infection result in the avirulence of the *ΔtmpL* mutants.

In conclusion, we have identified a novel transmembrane protein, TmpL, involved in plant and animal fungal virulence. Our results suggest that TmpL is involved in a complex redox homeostasis mechanism in *A. brassicicola* and *A. fumigatus* during fungal development and pathogenesis. Although the biochemical function of TmpL needs to be further investigated, it is plausible that the AMP-binding domain may activate signaling molecules and, together with the enzymatic activity generated by the FAD/NAD(P)-binding domain, regulate intracellular redox homeostasis. Since WBs have a peroxisomal origin [54], we can speculate that the TmpL protein, associated with WB, might also have a peroxisomal origin. Considering that peroxisomes play a key role in both the production and scavenging of ROS in the cell, H_2_O_2_ in particular [96], the peroxisome-originated TmpL may act as a detoxifier of ROS in the same way as many enzymatic peroxisomal membrane proteins and previously identified peroxisomal antioxidant regulators [97],[98]. Another recent finding to support this connection between fungal WB and oxidative stress is that disruption of *abhex1* in *A. brasscicola* resulted in mutant strains lacking WBs and were more sensitive to oxidative stress (H_2_O_2_) than wild-type (Kim et al., unpublished data). This result was meaningful because the deletion of *hex1* in other fungi also causes the complete loss of WB in the resulting mutants [76],[99]. Of further interest is that the *Δabhex1* mutants are not as hypersensitive as *ΔtmpL* strains, suggesting a possible, complicated relationship between the antioxidant involvement of the TmpL protein and its association with WB. Future studies will focus on identification of the specific substrate(s) directly or indirectly interacting with TmpL, and definitively determining the role of this interesting protein in plant and animal fungal virulence.

## Materials and Methods

### Fungal strains, media, fungal culture

*Alternaria brassicicola* strain ATCC 96866 was used in this study (American Type Culture Collection, Manassas, VA). The growth and maintenance of *A. brassicicola* and media composition were performed as described by Kim [37] except for a minimal medium (MM) (1% glucose, 0.5% (NH_4_)_2_SO_4_, 0.2% KH_2_PO_4_, 0.06% MgSO_4_, 0.06% CaCl_2_, 0.0005% FeSO_4_.7H_2_O, 0.00016% MnSO_4_.H_2_O, 0.00014% ZnSO_4_.7H_2_O, 0.00037% CoCl_2_.6H_2_O). *Aspergillus fumigatus* strain CEA10 was used as the wild-type, stored as frozen stock in 20% glycerol at −80°C, and grown at 37°C, on glucose minimal medium (GMM) with appropriate supplements as previously described [100] . *A. fumigatus* strain CEA17, a uracil auxotroph derived from CEA10, was used as the recipient strain for generation of the *ΔtmpL* mutant. In our study, solid complete medium (CM) refer to potato-dextrose agar and liquid CM to glucose-yeast extract broth (1% glucose, 0.5% yeast extract).

### Plant virulence assays

The virulence test on green cabbage (*Brassica oleracea*) was performed as described by Kim [37]. Briefly, *A. brassicicola* was inoculated with a 10 µl drop of conidial suspension (5×10^4^ or 20×10^4^ conidia ml^−1^) on each leaf of 5-week-old plants. Inoculated plants were kept in a plastic box at ambient temperatures and incubated at 100% humidity for 24 hr in the dark, followed by 16 hr fluorescent lights per day for 4–6 days. Lesion diameters were measured for all virulence tests. Statistical analyses were performed to test the differences in lesion diameters among the tested strains by a pairwise t-test using JMP software (SAS Institute Inc.). P-values≤0.01 were considered statistically significant. To test the ability of Ab*ΔtmpL* strain to colonize on wounded plants, the same conidial suspensions were applied to needle scratches on host plant leaves.

### Generation of *tmpL* replacement constructs, fungal transformation, and complementation in *A. brassicicola*

A *tmpL* replacement construct was made by a double-joint PCR method from three PCR fragments, with slight modifications [101]. Using *A. brassicicola* genomic DNA as a template, a 993 bp *tmpL* 5′ flanking region was amplified with primers TMPLR1 and TMPLR2 (Table S1), and a 992 bp *tmpL* 3′ flanking region was generated with primers TMPLR5 and TMPLR6. Using pCB1636 [102] as a template, a ∼1.4 kb hygromycin B phosphotransferase (*hph*) gene cassette was amplified with primers TMPLR3 and TMPLR4 . The reverse primer TMPLR2 that amplifies the 5′ flanking region and the forward primer ATMR5 that amplifies the 3′ flanking region, contained 20 bp tail sequences that overlapped the 5′ and 3′ ends of the *hph* cassette. Likewise, the forward and reverse primer TMPLR3 and TMPLR4 that amplified the *hph* cassette also contained a 20 bp tail sequences that overlapped the 5′ and 3′ flanking regions. The three PCR fragmentswere purified with the QIAquick PCR purification kit (Qiagen, Valencia, CA), were then diluted 10-fold, and subjected to fusion PCR with primers TMPLR1 and TMPLR6. The final 3.4 kb *tmpL* replacement construct was purified again with the QIAquick PCR purification kit and reduced to 1 µg/µl under vacuum before transformation. Fungal transformation was based on protocol described previously [56]. Transformants with expected genetic integrations were identified by PCR and Southern blot analysis.

In order to reintroduce wild-type *tmpL* into the *ΔtmpL* mutant, we amplified the wild-type *tmpL* allele from *A. brassicicola* genomic DNA using primer set TMPLcomF and TMPLcomR. The resulting PCR product covers 5.2 kb between the 953 bp upstream in relation to the start codon and the 1132 bp downstream in relation to the stop codon. A 1449 bp long nourseothricin resistance gene (*NAT*) cassette was amplified with primer set PNRcomF and PNRcomR from pNR1 plasmid [103]. The final two PCR products were used simultaneously to transform *ΔtmpL* mutant A1–3, and the transformants were selected using a nourseothricin antibiotic. PCR and Southern blot analyses were used to identify transformants with expected genetic integrations.

### Generation of *tmpL* replacement constructs, fungal transformation, and complementation in *A. fumigatus*

Generation of a *tmpL* null mutant in *A. fumigatus* strain CEA17 was accomplished by replacing an ∼1.9 kb internal fragment of the *tmpL* coding region (∼3.36 kb; GenBank accession no. EDP49089) with *A. parasiticus pyrG*. The disruption construct was generated by cloning a sequence homologous to the *tmpL* locus into plasmid pJW24 (donated by Nancy Keller, University of Wisconsin—Madison). The 5′ and 3′ *tmpL* homologous sequences, each ∼1 kb in length, were cloned to flank *A. parasiticus pyrG* in pJW24. The resulting plasmid, pTMPLKO, was used as a template to amplify the ∼5.2 kb disruption construct (primer RAC39 and RAC41) for use in fungal transformation. To complement the *ΔtmpL* strain, a plasmid with the *tmpL* gene connected to the *hph* gene was constructed. Therefore, the *tmpL* gene was amplified using genomic DNA of CEA10 as template and the primers RAC357 and RAC110. The ∼5.3 kb PCR product and the plasmid pBC-Hygro were digested with *Not*I and *Spe*I. The PCR product was then ligated into the vector. The resulting plasmid, pTMPLREC, was used as a template to amplify the ∼9.5 kb reconstitution construct (primer RAC325 and RAC326) for use in fungal transformation.

Generation of fungal protoplasts and polyethylene glycol-mediated transformation of *A. fumigatus* were performed as previously described [104]. Briefly, 10 µg of the *tmpL*KO PCR-generated disruption construct was incubated on ice for 50 min with 1×10^7^ fungal protoplasts in a total volume of 100 µl. Gene disruption transformants were initially screened by PCR to identify potential homologous recombination events at the *tmpL* locus. PCR was performed with primers designed to amplify only the disrupted *tmpL* locus - RAC109 and RAC22 (PCR product: 2077 bp); RAC21 and RAC110 (PCR product: 1595 bp). For the reconstituted strain, 10 µg of the *tmpL*REC PCR-generated reconstitution construct was used in the protoplast transformation. Colonies were selected for growth on hygromycin containing media. Reconstitution events were then screened by PCR by amplifying a part of the *tmpL* that was replaced by *pyrG* in the mutant [RAC351 and RAC352 (PCR product: 778 bp)]. Homologous recombination of the disruption cassette and random integration of the reconstituition construct was confirmed by Southern analysis with the digoxigenin labeling system (Roche Molecular Biochemicals, Mannheim, Germany) as previously described [105]. To eliminate the chance of heterokaryons, each transformant was streaked with sterile toothpicks a minimum of two times to obtain colonies from single conidia.

### Preparation of *A. brassicicola* nucleic acids

DNA isolation and Southern blot analysis were performed as described by Kim [37]. The *tmpL* 3′ fragment was used as an *tmpL* specific probe and a 500 bp *hph* fragment from the pCB1636 plasmid was used as a *hph* specific probe, and a ∼1 kb *NAT* fragment from pNR1 plasmid as a *NAT* specific probe. All sequencing was done using the ABI Prism 310 automated sequencer (Applied Biosystems, Forster City, CA). Total RNA was extracted from fungal samples using the RNeasy Plant Kit according to the manufacturer's protocol (Qiagen, Valencia, CA). For the expression analysis with QRT-PCR, leaves of green cabbage were inoculated with 10 µl drops of wild-type conidial suspension (1×10^7^ conidia ml^−1^), and infected samples were collected at 12, 24, 48, 60, 72, 96, and 120 hr after inoculation. Total RNA was also extracted from mycelia grown in liquid CM for 72 hr. In order to maintain vegetative growth with no stress, the liquid CM was changed every 24 hr. About 20 mycelial balls collected from the above 72 hr-liquid culture were spread onto sterilized filter paper, incubated for conidiation, and collected at 24 and 48 hr for total RNA extraction.

### Expression and purification of TmpL FAD/NAD(P)-binding region and FAD-binding assay

First-strand cDNA was generated from the total RNA of 48 hr air-exposed mycelial balls with random primers using SuperScript™ First-Strand Synthesis System (Invitrogen™ Life Technologies, Carlsbad, CA, USA). A 635 bp *tmpL* partial coding sequence containing the FAD/NAD(P)-binding region was amplified from the cDNA using primers A1fn_ExpKpnFor and A1fn_ExpHndRev, and cloned between the *Kpn*I and *Hind*III sites in plasmid pKLD66 [106] to obtain plasmid pA1FN. *E. coli* BL21(DE3) was transformed with pA1FN. *E. coli* BL21(DE3)(pA1FN) was grown to an optical density of 0.6–0.8, followed by induction with 0.2 mM IPTG. After 3 hr of induction, cells were harvested by centrifugation at 7000×g for 10 min at 4°C. The resulting 2 g cell pellet was resuspended in 2.5 ml nickel-nitrilotriacetic acid (Ni-NTA) 50 mM sodium phosphate buffer, pH 7.5. The cell suspension was passed three times through a French pressure cell at a pressure of 1.28×10^8^ Pa. The resulting cell lysate was centrifuged at 8000×g for 25 min at 4°C to remove cell debris. The resulting supernatant was mixed with l ml Ni-NTA His Bind Resin (Novagen) and incubated for 1 hr at 4°C with constant agitation. The incubated solution was loaded onto a column bed and the column was washed with 10 ml Ni-NTA washing buffer (50 mM sodium phosphate (pH 7.5) and 20 mM imidazole). The column was sequentially eluted with 50–500 mM imidazole containing 50 mM sodium phosphate buffer (pH 7.5). Fractions at about 250 mM imidazole were pooled and concentrated on an YM-30 membrane (Amicon). The 1 mg protein concentrate was incubated with 0.2 mM FAD at 4°C for 5 hr. Free flavin was removed by filtration and three 1 mL washes with 50 mM sodium phosphate buffer (pH7.5), on the membrane of a YM-3 concentrator (Amicon). The product was recovered in 50 mM sodium phosphate buffer (pH7.5) and assayed for protein content. A UV-visible spectrum of the protein was analysed with 200–800 nm wavelength range.

### Generation of fusion protein constructs

A *tmpL* C-terminal *gfp* fusion construct was generated by fusion PCR. Using *A. brassicicola* genomic DNA as a template, an 1 kb *tmpL* 3′ region was amplified with primers TMPLGFP1 and TMPLGFP2-GA. Another set of primers, TMPLGFP3-GA and TMPLGFP4, were used to amplify a 2.4 kb *gfp* and *hph* cassette from template plasmid pCB16G6-Nac [56]. Two resulting fragments, the 1 kb *tmpL* 3′ fragment and the 2.4 kb *gfp* and *hph* cassette, were mixed and subjected to second fusion PCR with primers TMPLGFP1 and TMPLGFP4. The resulting 3.4 kb PCR products were transformed in the *A. brassicicola* wild-type to make TmpL-GFP fusion transformants. Transformants with expected genetic integration events were identified by PCR and Southern blot analyses.

The same fusion PCR strategy was applied to generate a series of fusion proteins in which different portions of *tmpL*, an AMP-binding and transmembrane domain, were appended to the N terminus of the *gfp*. For the *tmpL* AMP-binding-*gfp* fusion construct, two primers, A1AdeGFP1 and A1AdeGFP2-GA, were used to amplify an 881 bp *tmpL* AMP-binding domain region. Another set of primers, A1AdeGFP3-GA and A1AdeGFP4, were used to amplify a 2.4 kb *gfp* and *hph* cassette from template plasmid pCB16G6-Nac. The two resulting PCR fragments were subjected to second fusion PCR with primers A1AdeGFP1 and A1AdeGFP4. The resulting 3.4 kb PCR products were transformed in the wild-type. In the same way, four primers were designed to generate the *tmpL* transmembrane-*gfp* fusion construct as follows: A1TmGFP1 and A1TmGFP2-GA for a 756 bp *tmpL* transmembrane region; A1TmGFP3-GA and A1TmGFP4 for a *gfp* and *hph* cassette.

To generate the *DsRed*-*abhex1* fusion construct by fusion PCR, three PCR fragments were amplified as follows: a 573 bp *Pyrenophora tritici- repentis ToxA* promoter fragment using primers ToxAFor and ToxA-DsRedRev from template plasmid pCB16G6-Nac; a 728 bp *DsRed* ORF fragment using primers DsRed-ToxAFor and DsRed-AbHEX1Rev from template plasmid pCAG-DsRed [107]; a 969 bp *abhex1* fragment using primers AbHEX1-DsRedFor and AbHEX1Rev from *A. brassicicola* genomic DNA. These final three PCR fragments were subjected to second fusion PCR with primers ToxAFor and AbHEX1Rev. The final construct was transformed into the TmpL-GFP fusion strain to generate TmpL-GFP:DsRed-AbHex1 dual fluorescence-labeled strains.

To construct the *DsRed*-PTS1 construct that serves as marker of peroxisomal matrix, the *DsRed* fragment was amplified from pCAG-DsRed plasmid using primers DsRedPTS1For and DsRedPTS1Rev, which append the PTS1 tripeptide SRL to the C terminus of *DsRed*. Using pNR1 as template, a 1.4 kb nourseothricin resistance gene (*NAT*) cassette was amplified with primers DsRedPTS1NATFor and DsRedPTS1NATRev. These final two PCR fragments were subjected to second fusion PCR with primers DsRedPTS1For and DsRedPTS1NATRev. The final construct was transformed into the TmpL-GFP strain to generate TmpL-GFP:DsRed-PTS1 dual fluorescence-labeled strains.

To disrupt *pex14* in TmpL-GFP and DsRed-AbHex1 strains, a linear minimal element (LME) construct was generated as previously described [56]. Primers pex14KOFor and pex14KORev were used to amplify a 415 bp *pex14* partial fragment from the *A. brassicicola* genomic DNA and another set of two primers, pex14HygFor and pex14HygRev, were used to amplify an 1.4 kb *NAT* cassettes from the plasmid pNR1. The two fragments were subjected to second fusion PCR with primers pex14KOFor and pex14HygRev. The final construct was transformed into the TmpL-GFP and DsRed-AbHex1 strains to generate TmpL-GFP:*Δpex14* and DsRed-AbHex1:*Δpex14* mutant strains, respectively.

To generate *gfp*-*yap1* construct under the control of the *yap1* promoter, four PCR fragments were amplified by fusion PCR. A 500 bp fragment of the *yap1* promoter region was produced from *A. brassicicola* genomic DNA using primers PromoYap1For and PromoYap1Rev, a 570 bp fragment of the *gfp* ORF region from pCB16G6-Nac plasmid using primers GFPYap1For and GFPYap1Rev, an 1 kb *yap1* ORF from the genomic DNA using primers Yap1For and Yap1Rev, and an 1.4 kb *NAT* cassette from plasmid pNR1 using primers Yap1NATFor and Yap1NATRev. These four fragments were subjected to second fusion PCR with primers PromoYap1For and Yap1NATRev. The final construct was transformed into the wild-type and *A. brassicicola ΔtmpL* mutant.

To generate *ToxA*-*yap1* overexpression construct, a 400 bp fragment of the *ToxA* promoter region from pCB16G6-Nac plasmid using primers ToxAFor and ToxAYap1Rev, and a 3.4 kb *yap1* and *NAT* cassette from the above *gfp*-*yap1* construct using primers Yap1overFor and Yap1NATRev were subjected to second fusion PCR with primers ToxAFor and Yap1NATRev. The final construct was transformed into wild-type and the *A. brassicicola ΔtmpL* mutant.

All construct were subject to sequence verification with the ABI Prism 310 automated sequencer (Applied Biosystems, Forster City, CA). All transformants with expected genetic integration events were identified by PCR and Southern blot analysis.

### Quantitative real-time PCR

To analyze the mRNA abundance of *tmpL* by quantitative real-time (QRT) PCR, 1 µg of total RNA was used for first-strand cDNA with random primers using SuperScript™ First-Strand Synthesis System (Invitrogen™ Life Technologies, Carlsbad, CA, USA) according to the manufacturer's instruction and diluted 1∶3 with nuclease-free water. Reactions were performed in a 25 µl volume containing 100 nM of each primer, 2 µl of cDNA (25 ng of input RNA) and 12.5 µl of 2X iQ™ SYBR® Green Supermix (Bio-Rad, Hercules, CA, USA). QRT-PCR was run on the iCycler iQ Real-Time PCR Detection System (Bio-Rad, Hercules, CA, USA). After a 3 min denaturation at 95°C, samples were run for 40 cycles of 15 s at 95°C, 30 s at 60°C and 30 s at 72°C. After each run, amplification specificity was checked with a dissociation curve acquired by heating the samples from 60 to 95°C. To compare relative abundance of *tmpL* transcripts, average threshold cycle (Ct) was normalized to that of Glyceraldehyde-3-phosphate dehydrogenase (*GAPDH*) for each condition as 2^−ΔCt^, where −ΔCt = (C_t_,*_tmpL_*−C_t_,*_GAPDH_*). Fold changes during conidial development and during infectious growth compared with growing fungus in liquid CM were calculated as 2^−ΔΔCt^, where −ΔΔCt = (C_t_,*_tmpL_*−C_t_,*_GAPDH_*)_test condition_−(C_t_,*_tmpL_*−C_t_,*_GAPDH_*)_liquid_ [108]. The same real-time PCR strategy was used to analyze the expression of *yap1* and other antioxidant-related genes in *A. brassicicola* wild-type, WT:pToxAYap1 mutant, Ab*ΔtmpL*, and Ab*ΔtmpL*:pToxAYap1 strains, except for the method of calculating relative fold change. It was determined by comparing each expression level with the one of vegetatively growing wild-type in liquid CM, where −ΔΔCt = (C_t_,_gene of interest_−C_t_,*_GAPDH_*)_rest conditions_−(C_t_,_gene of interest_−C_t_,*_GAPDH_*)_WT,vegetative mycelia_. Each QRT-PCR was conducted twice with two replicates and all the data is presented. The primer pairs for the transcript amplification of each gene were as follows: For the *tmpL* gene, TMPL-expFor and TMPL-expRev; *yap1*, Yap1-expFor and Yap1-expRev; *skn7*, SKN7-expFor and SKN7-expRev; *ctt1*, CTT1-expFor and CTT1-expRev; *sod1*, SOD1-expFor and SOD1-expRev; *gsh1*, GSH1-expFor and GSH1-expRev; *gsh2*, GSH2-expFor and GSH2-expRev; *trx2*, TRX2-expFor and TRX2-expRev; *gpx1*, GPX1-expFor and GPX1-expRev. For amplification of the internal control *GAPDH* gene, AbGAPDH-For and AbGAPDH-Rev were used.

### Oxidative stress assays

For the oxidative stress tests, *A. brassicicola* and *A. fumigatus* were grown on solid MM with or without the stress agents KO_2_ and H_2_O_2_. Sensitivity to each stressor was determined by comparing the colony radius of 5-day-old *A. brassicicola* cultures on media containing each stressor. The tests were repeated at least three times for each condition. For the germling susceptibility assay in *A. fumigatus*, a protocol from the laboratory of Judith Rhodes University of Cincinnati was followed. Briefly, conidia from CEA10, Af*ΔtmpL* and AftmpL rec were harvested after growth on GMM plates for 3 days and incubation at 37°C. The conidia were diluted and counted in a hemocytometer. The strains were adjusted to 200 colonies per plate when 100 µl was plated. The strains were challenged in triplicate on GMM plates with 1.25mM H_2_O_2_, plus the control. The plates were incubated at 30°C until microscopic germlings appeared on the plates (about 16 hrs). Then the plates were overlaid with 10 ml of 1.25mM H_2_O_2_ or 10 ml distilled water as a control and incubated at 37°C for 10 minutes. After aspirating off the H_2_O_2_ and washing the plate twice with 10 ml of sterile distilled water the plates were returned to the 30°C incubator and incubated until colonies were large enough to count.

### Murine virulence assays

In this study, an outbred CD1 (Charles River Laboratory, Raleigh, NC) strain was used. All animals were kept in specific pathogen-free housing, and all animals were handled in strict accordance with good animal practice as defined by the relevant national and/or local animal welfare bodies, and all animal work was approved by the appropriate institutional internal review board (IACUC) committee. Male mice (26 to 28 g in size, 6–8 weeks old), were housed five per cage and had access to food and water ad libitum. Mice were immunosuppressed with intraperitoneal (i.p.) injections of cyclophosphamide at 150 mg/kg 3 days prior to infection and with Kenalog injected subcutaneously (s.c.) at 40 mg/kg 2 days prior to infection. On day 3 post-infection (p.i.), repeat injections were given with cyclophosphamide (150 mg/kg i.p.) and on day 6 p.i. with Kenalog (40 mg/kg s.c.). Ten mice per A. fumigatus strains (CEA10, tmpL-deficient mutant, or the reconstituted strain AftmpL rec) were infected intranasally. The mice were inoculated intranasally following brief isoflurane inhalation, returned to their cages, and monitored at least twice daily.

Infection inoculum was prepared by growing the *A. fumigatus* isolates on GMM agar plates at 37°C for 3 days. Conidia were harvested by washing the plate surface with sterile phosphate-buffered saline-0.01% Tween 80. The resulting conidial suspension was adjusted to the desired concentration of 1×10^6^ conidia/25 µl by hemacytometer count. Mice were observed for survival for 14 days after *A. fumigatus* challenge. Any animals showing distress were immediately sacrificed and recorded as deaths within 24 hr. Mock mice were included in all experiments and inoculated with sterile 0.01% Tween 80. Survival was plotted on a Kaplan-Meier curve and a log-rank test used to determine significance of pair-wise survival (two-tailed P<0.01). The animal experiments were repeated on two separate occasions with similar results.

### Histopathology

For histopathology studies, additional CD1 mice were infected with the wild-type CEA10, *tmpL* mutant, or tween/saline control as described for virulence studies, and 3 mice were sacrificed at set time points of day 2 and day 4 after *A. fumigatus* challenge. When mice were sacrificed, lungs were immediately removed on that day. Lung tissue was fixed in 10% phosphate-buffered formalin, embedded in paraffin, sectioned at 5 µm, and stained with hematoxylin and eosin (H&E) or Grocott methenamine silver (GMS) by using standard histological techniques. Microscopic examinations were performed on a Zeiss Axioscope 2-plus microscope and imaging system using Zeiss Axiovision version 4.4 software.

### Microscopy

For confocal microscopy, an inverted confocal laser scanning microscope (LSM-510, Carl Zeiss, Göttingen, Germany) and an argon ion laser for excitation at 488 nm wavelength and GFP filters for emission at 515–530 nm were used. Transformants carrying each fluorescent protein fusion construct were grown on solid and liquid CM. Newly formed conidia and conidiophores from solid CM plates and vegetative mycelia from liquid CM were collected for viewing. For *in planta* expression analysis, the lower epidermis of green cabbage cotyledons was peeled off at 4 and 12 hpi and observed. For the DsRed fusion strains, a He-Ne laser (543 nm excitation, 560–615 nm emission) was used. The imaging parameters used produced no detectable background signal from any source other than from each fluorescent protein. Confocal images were captured with LSM-510 software (version 3.5; Carl Zeiss) and recorded simultaneously by phase contrast microscopy and fluorescence confocal microscopy. Brightfield and DIC images were captured with a photomultiplier for transmitted light using the same laser illumination for fluorescence.

For the electron microscopy, conidia from each strain were released in sterile water and processed as described previously for transmission electron microscopy [37]. Examination was conducted with a JEM-1010 transmission electron microscope (JEOL, Tokyo, Japan) operating at 60 kV. For cross-sections of the green cabbage leaves inoculated with *A. brassicicola* wild-type and *ΔtmpL* strains, leaf samples were collected, embedded in Epon resin, cut thick sections with an ultramicrotome (MT-X, RMC, USA), and stained with 1% toluidine blue O. The thick sections were observed using a light microscope (Eclipse E600; Nikon, Tokyo, Japan).

### Cytological assays

For cytological analysis, the lower epidermis of green cabbage cotyledons was peeled off 12 hpi, stained with lactophenol-cotton blue [109], and observed by light microscopy. For the onion epidermis assay, the epidermis was peeled off, carefully washed with distilled water,then inoculated with the conidia on the adaxial surface. After 12, 24, 48, and 72 hr incubation in a closed Petri dish at 100% RH, the epidermis was stained with lactophenol-cotton blue and observed by light microscopy.

For the detection of callose papillae, green cabbage cotyledons inoculated with *A. brassicicola* were fixed and decolorized in boiling 95% ethanol, then stained in aniline blue (0.005% (w/v) in 0.07 M K_2_HPO_4_). Callose was observed by mounting stained tissue in 70% glycerin and water viewing on an Axioplan Universal microscope (Carl Zeiss Microscope Division, Oberkochen, Germany) with a fluorescein filter set with excitation at 365 nm and emission at 420 nm.

ROS was detected by staining with following solutions. For superoxide detection, nitroblue tetrazolium (Sigma-Aldrich) was used at 5 mg·ml^−1^ and the staining performed for 1 hr at room temperature prior to observation. *A. brassicicola* conidia collected from a nutrient-rich medium (5 g yeast extract, 5 g casamino acid, 340 g sucrose, 15 g agar in 1 L deionized water) and fungus-inoculated leaves at each time point were subjected to the staining. For detection of ROS other than superoxide, *A. brassicicola* and *A. fumigatus* conidia were collected from PDA and GMM media, respectively, and stained with 5 µg·ml^−1^ 5-(and 6)-carboxy-2′,7′-dichlorodihydrofluorescein diacetate (carboxy-H_2_DCFDA; Molecular Probes, Eugene, OR). The intracellular distribution of ROS in appressoria was visualized after staining with 2 mg/ml DAB 2 hr, followed by a short rinse PBS.

### Accession numbers

Sequence data for *tmpL* can be found in the GenBank data libraries under accession number EU223383 for *A. brassicicola* and EDP49089 for *A. fumigatus*.

## Supporting Information

Figure S1 Phylogenetic analysis of the TmpL orthologs. TmpL orthologs were aligned with ClustalW, the alignment imported into PFAAT, and a neighbor joining tree generated based on its predicted amino acid sequences. Numbers at the node represent the result of 100 bootstrap replications. GenBank or organism-specific accession numbers follow species abbreviations. Red indicates Dothideomycetes, green for Sordariomycetes, yellow for Homobasidiomycota, and blue for Eurotiomycetes. Abbreviations: Pn *Phaeosphaeria nodorum*, Ch *Cochliobolus heterostrophus*, Ab *Alternaria brassicicola*, Fv *Fusarium verticillioides*, Fo *Fusarium oxysporum*, Gz *Gibberella zeae*, Cc *Coprinopsis cinerea*, Pc *Phanerochaete chrysosporium*, Mg *Magnaporthe grisea*, Af *Aspergillus fumigatus*, Nf *Neosartorya fischeri*, At *Aspergillus terreus*, Ac *Aspergillus clavatus*, An *Aspergillus nidulans*.
(0.08 MB PDF)

Figure S2 Sequence comparison of *A. brassicicola* TmpL protein with *Aspergillus nidulans* TmpA protein. The partial amino acid sequence (501–1025) of TmpL is aligned with TmpA protein (GenBank accession no. AAP13095) from *A. nidulans* using ClustalW2. Identical amino acid residues are indicated by asterisks and similar residues by dots. Predicted transmembrane domains in TmpL and TmpA are underlined. Hypothetical FAD (RLHFD) and NAD(P) (GSGIGP) phosphate-binding domains are indicated with light and dark gray boxes respectively.
(0.03 MB PDF)

Figure S3 Ultrastructure of Woronin bodies and epifluorescence microscopy of conidiophore of TmpL-GFP mutant strain. (A) Transmission electron micrographs of *A. brassicicola* wild-type conidia showing Woronin body localization. Note that there are two locations of Woronin bodies: near septal pores (arrowheads) and apart from the septal pores, in cytoplasm (arrows). This supports findings of the confocal microscopy of TmpL-GFP and DsRed-AbHex1 localization assay. Bars = 500 nm. Abbreviations: L, lipid body; M, mitochondria; P, peroxisome; S, septa; V, vacuole. (B) Epifluorescence microscopy of the TmpL-GFP mutant strain showed that *tmpL* is highly expressed in conidiophores. Bar = 500 nm.
(0.56 MB PDF)

Figure S4 Targeted gene replacement of the *A. brassicicola tmpL* locus. (A) A gene replacement construct was generated by fusion PCR method and used for transformation of fungal protoplasts of *A. brassicicola* isolate ATCC96866. Shown are wild-type *tmpL* gene locus, a replacement cassette, and Ab *ΔtmpL* mutant locus replaced by the cassette. The mutated genomic locus of Ab *ΔtmpL* mutant is depicted to show homologous recombination of the replacement cassette. (B) *A. brassicicola* wild-type (WT), ectopic mutant (A1E1), and replacement of wild-type *tmpL* with a single copy of *hph* cassette by homologous recombination in two Ab *ΔtmpL* mutants (A1–3 and A1–4) were first screened by PCR with primers (P1/P2). (C) Southern blot analysis of *A. brassicicola* wild-type strain (WT), ectopic mutant (A1E1), two *ΔtmpL* mutants (A1–3 and A1–4), and two reconstituted mutants (A1C1 and A1C2). The wild-type and a hygromycin-resistant mutant A1E1 both contained a 2.5 kb *Bsr*GI fragment, but Southern blotting with *hph* fragment showed a 5 kb band in A1E1 (data not shown), indicating ectopic integration of a possible truncated replacement construct. A band shift to about 10 kb was detected in both Ab *ΔtmpL* mutants, indicating that homologous recombination occurred at a single site. The complemented mutants, A1C1 and A1C2, generated from a mutant strain A1–3 showed the same 2.5 kb band to the wild-type, indicating Ab *ΔtmpL* mutant A1–3 have been complemented by a full-length *tmpL* gene fragment. The letter B on the genomic locus (A) indicates enzymatic sites for *Bsr*GI that were used of genomic DNA digestion. The region used for labeling the hybridization probe is marked with a bar under the replacement cassette. (D) Reverse transcription (RT)-PCR showing *tmpL* transcripts from mycelia actively producing conidia of *A. brassicicola* wild-type (WT), ectopic mutant (A1E1), two *ΔtmpL* mutants (A1–3 and A1–4), and reconstituted mutant (A1C2). RT-PCR showed that *tmpL* transcripts are not detected among Ab *ΔtmpL* mutants during active conidiogenesis when mycelia were exposed to ambient air. *actin* was used as an internal control.
(0.08 MB PDF)

Figure S5 Targeted gene replacement of the *A. fumigatus tmpL* locus. (A) *A. fumigatus* wild-type *tmpL* gene locus was replaced by the *A. parasiticus pyrG* cassette, resulting in Af *ΔtmpL* mutant locus. The mutated genomic locus of Af *ΔtmpL* mutant is depicted to show homologous recombination of the replacement cassette. (B) Southern blot analysis of *A. fumigatus* wild-type strain (CEA10), *ΔtmpL* mutant (Af *ΔtmpL*), and reconstituted strain (AftmpL rec). The wild-type strain CEA10 contained a 6.3 kb *Nco*I fragment. A band shift to about 3.8 kb was detected in Af *ΔtmpL* strain, indicating that homologous recombination occurred at a single site. The reconstituted strain AftmpL rec showed the same 6.3 kb band to the wild-type and 3.8 kb band to the Af *ΔtmpL* strain, indicating the Af *ΔtmpL* mutant has been ectopically complemented by a full-length *tmpL* gene fragment. The letter N on the genomic locus (A) indicates enzymatic sites for *Nco*I that were used of genomic DNA digestion. The region used for labeling the hybridization probe is marked with a bar (Probe).
(0.06 MB PDF)

Figure S6 Detection of cell death in *A. brassicicola* wild-type and *ΔtmpL* conidia stained with annexin V-FITC. (A) Conidia collected from 7- and 21-day-old fungal colonies grown on solid CM were subjected to annexin V-FITC staining. Percentage of conidia showing fluorescence that are classified as dead cells was measured. Columns and error bars represent average and SD, respectively, of two independent experiments. (B) Representative micrographs showing an annexin V-FITC positive conidial cell of the 21-day-old Ab *ΔtmpL* mutant, while no staining in the 21-day-old wild-type conidia.
(0.25 MB PDF)

Figure S7 Formation of appressoria and infection hyphae and a virulence assay of wounded and non-wounded green cabbage leaves inoculated with *A. brassicicola* wild-type and *ΔtmpL* mutant. (A) Green cabbage cotyledons were used to examine the appressoria and infection hyphae formation of wild-type and Ab *ΔtmpL* mutant infection. Intracellular infection hyphae of the Ab *ΔtmpL* mutant were rarely developed inside of the plant epidermal cells, while infection hyphae of the wild-type appressoria were consistently observed. Bars = 20 µm. Abbreviations: a, appressorium; ih, infection hypha. (B) Green cabbage leaves inoculated with wild-type and Ab *ΔtmpL* mutant were collected at 12 and 24 hpi, embedded epoxy resin, sectioned, and stained with 0.1% toluidine blue O. Due to the massive secretion of fungal enzymes and toxins from the appressoria and infection hyphae of the wild-type, plant tissues around the fungal cells were extensively macerated and degraded and plastids were abnormally inflated (arrows). By contrast, leaf sections inoculated with the Ab *ΔtmpL* mutant maintained almost intact plant tissue (12 hpi) and plant cells below the infection site showed cell necrosis or callose-deposition-like phenomenon at 24 hpi (arrowheads). Bars = 20 µm. Abbreviations: a, appressorium; ec, epidermal cell; ih, infection hypha. (C) Wounded leaf infection assay of wild-type and Ab *ΔtmpL* mutant. The upper panel indicates intact (non-wounded) leaf inoculated with the wild-type and Ab *ΔtmpL* mutant, and the lower panel depicts wounded leaf infection by needle scratching. The Ab *ΔtmpL* mutant formed larger lesions on wounded leaves compared with the tiny lesions on intact leaves but were still smaller than those resulting from wild-type inoculations on wounded leaves.
(0.37 MB PDF)

Table S1 Primer sequences used in this study.
(0.02 MB PDF)
