# Supplementary figures and images for: TmpL, a Transmembrane Protein Required for Intracellular Redox Homeostasis and Virulence in a Plant and an Animal Fungal Pathogen
Source: PLoS Pathog. 2009 Nov 6;5(11):e1000653. doi: 10.1371/journal.ppat.1000653 (PMC2766074; doi:10.1371/journal.ppat.1000653)

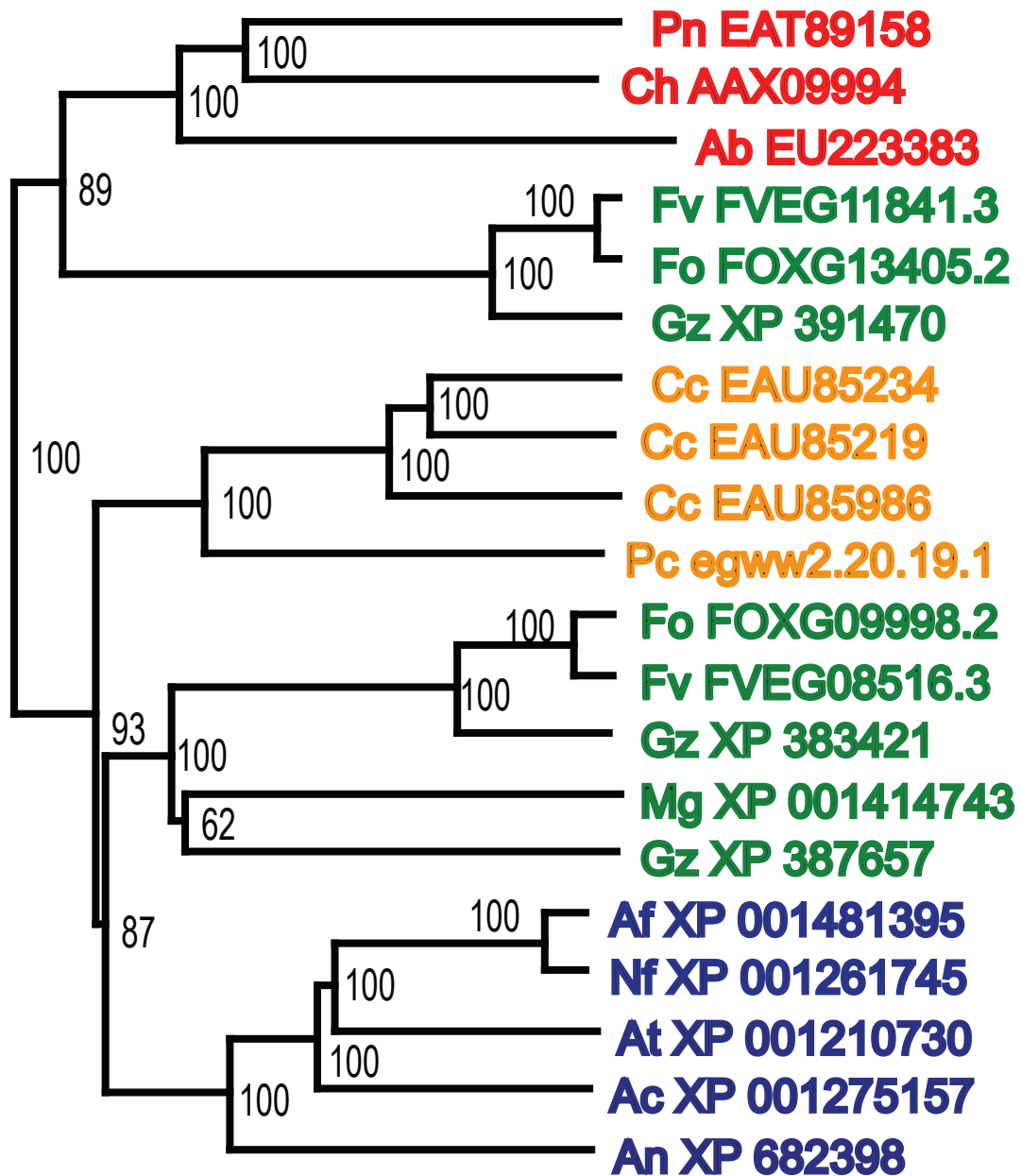

Supplement: Figure S1 — Phylogenetic analysis of the TmpL orthologs. TmpL orthologs were aligned with ClustalW, the alignment imported into PFAAT, and a neighbor joining tree generated based on its predicted amino acid sequences. Numbers at the node represent the result of 100 bootstrap replications. GenBank or organism-specific accession numbers follow species abbreviations. Red indicates Dothideomycetes, green for Sordariomycetes, yellow for Homobasidiomycota, and blue for Eurotiomycetes. Abbreviations: Pn Phaeosphaeria nodorum, Ch Cochliobolus heterostrophus, Ab Alternaria brassicicola, Fv Fusarium verticillioides, Fo Fusarium oxysporum, Gz Gibberella zeae, Cc Coprinopsis cinerea, Pc Phanerochaete chrysosporium, Mg Magnaporthe grisea, Af Aspergillus fumigatus, Nf Neosartorya fischeri, At Aspergillus terreus, Ac Aspergillus clavatus, An Aspergillus nidulans. (0.08 MB PDF) [file ppat.1000653.s001.pdf]

**A**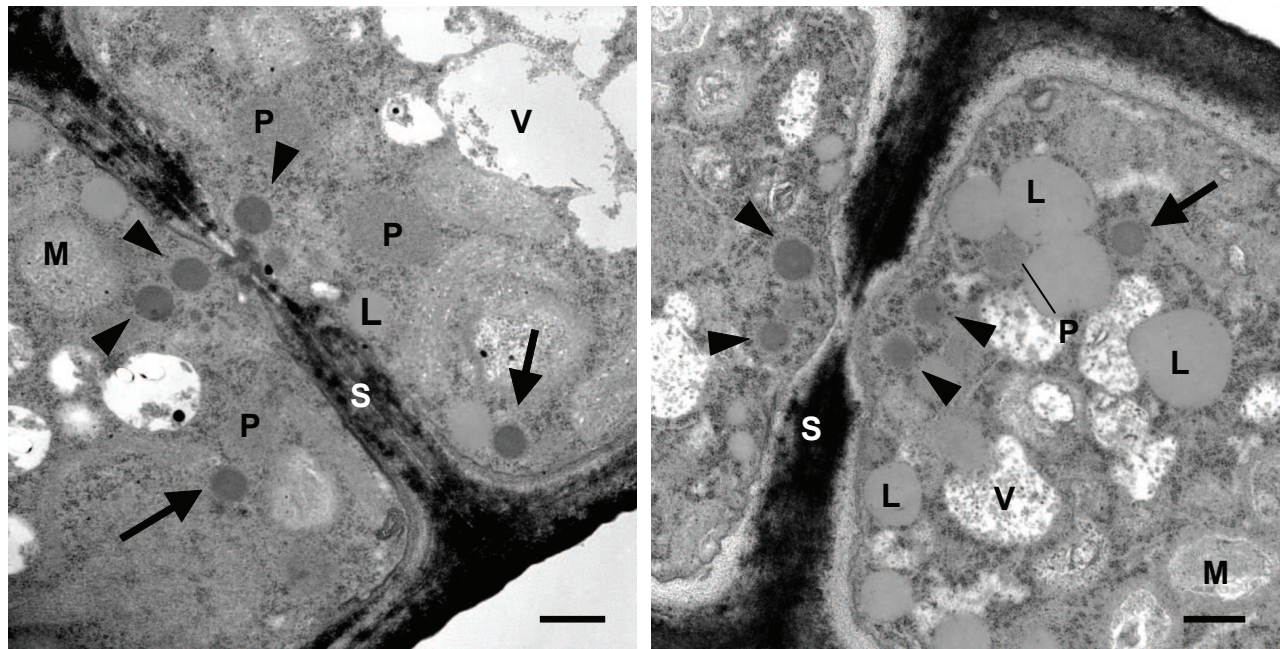**B**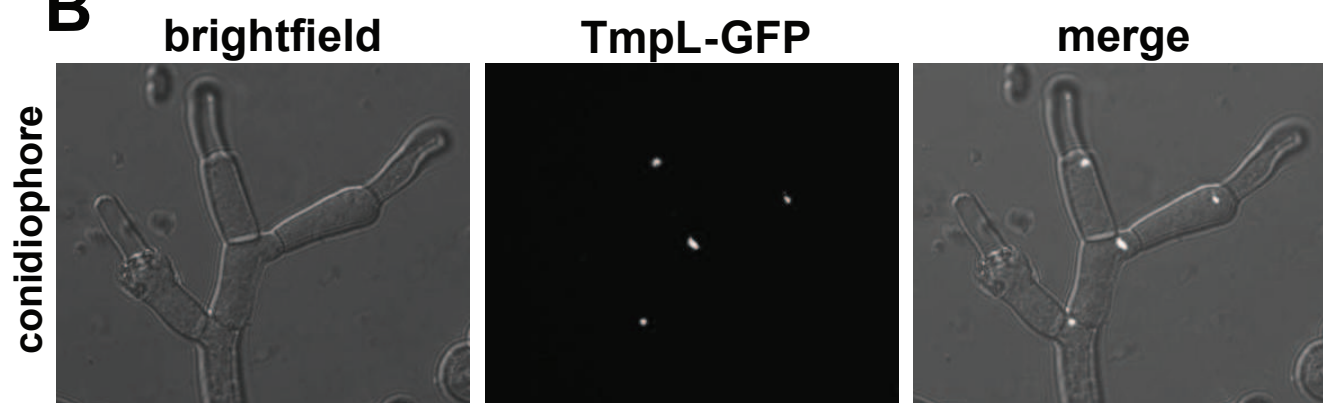

Supplement: Figure S3 — Ultrastructure of Woronin bodies and epifluorescence microscopy of conidiophore of TmpL-GFP mutant strain. (A) Transmission electron micrographs of A. brassicicola wild-type conidia showing Woronin body localization. Note that there are two locations of Woronin bodies: near septal pores (arrowheads) and apart from the septal pores, in cytoplasm (arrows). This supports findings of the confocal microscopy of TmpL-GFP and DsRed-AbHex1 localization assay. Bars = 500 nm. Abbreviations: L, lipid body; M, mitochondria; P, peroxisome; S, septa; V, vacuole. (B) Epifluorescence microscopy of the TmpL-GFP mutant strain showed that tmpL is highly expressed in conidiophores. Bar = 500 nm. (0.56 MB PDF) [file ppat.1000653.s003.pdf]

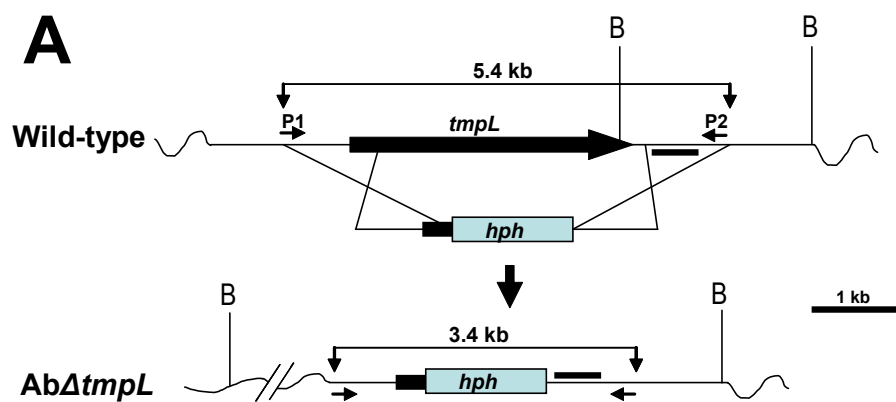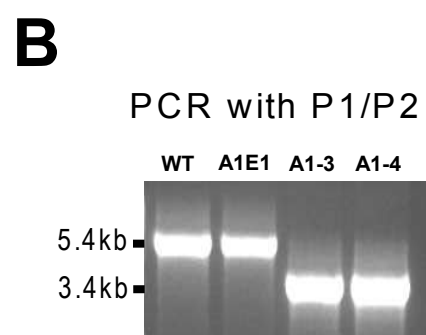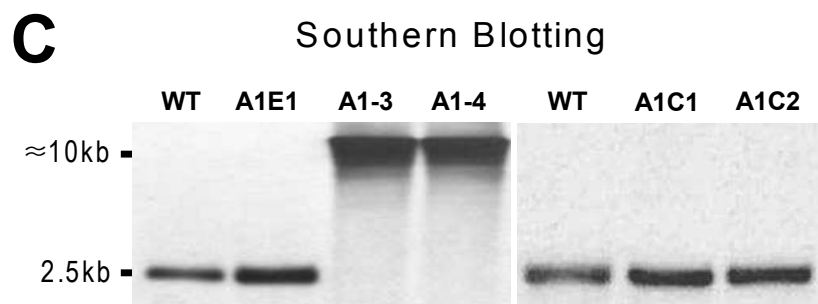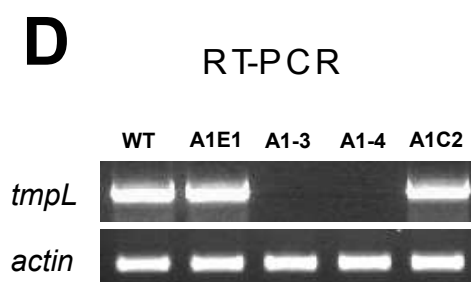

Supplement: Figure S4 — Targeted gene replacement of the A. brassicicola tmpL locus. (A) A gene replacement construct was generated by fusion PCR method and used for transformation of fungal protoplasts of A. brassicicola isolate ATCC96866. Shown are wild-type tmpL gene locus, a replacement cassette, and Ab ΔtmpL mutant locus replaced by the cassette. The mutated genomic locus of Ab ΔtmpL mutant is depicted to show homologous recombination of the replacement cassette. (B) A. brassicicola wild-type (WT), ectopic mutant (A1E1), and replacement of wild-type tmpL with a single copy of hph cassette by homologous recombination in two Ab ΔtmpL mutants (A1–3 and A1–4) were first screened by PCR with primers (P1/P2). (C) Southern blot analysis of A. brassicicola wild-type strain (WT), ectopic mutant (A1E1), two ΔtmpL mutants (A1–3 and A1–4), and two reconstituted mutants (A1C1 and A1C2). The wild-type and a hygromycin-resistant mutant A1E1 both contained a 2.5 kb BsrGI fragment, but Southern blotting with hph fragment showed a 5 kb band in A1E1 (data not shown), indicating ectopic integration of a possible truncated replacement construct. A band shift to about 10 kb was detected in both Ab ΔtmpL mutants, indicating that homologous recombination occurred at a single site. The complemented mutants, A1C1 and A1C2, generated from a mutant strain A1–3 showed the same 2.5 kb band to the wild-type, indicating Ab ΔtmpL mutant A1–3 have been complemented by a full-length tmpL gene fragment. The letter B on the genomic locus (A) indicates enzymatic sites for BsrGI that were used of genomic DNA digestion. The region used for labeling the hybridization probe is marked with a bar under the replacement cassette. (D) Reverse transcription (RT)-PCR showing tmpL transcripts from mycelia actively producing conidia of A. brassicicola wild-type (WT), ectopic mutant (A1E1), two ΔtmpL mutants (A1–3 and A1–4), and reconstituted mutant (A1C2). RT-PCR showed that tmpL transcripts are not detected among Ab ΔtmpL mutants dur [file ppat.1000653.s004.pdf]

**A****Wild-type locus**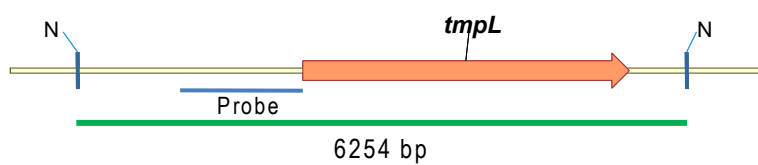***AfΔtmpL* locus**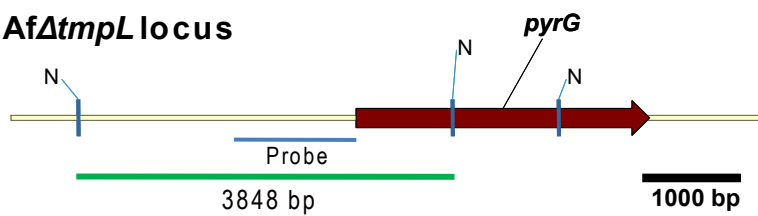**B****Southern Blotting**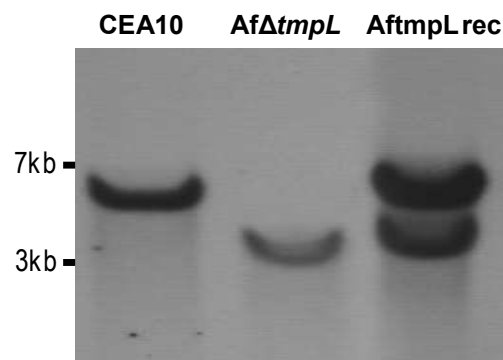

Supplement: Figure S5 — Targeted gene replacement of the A. fumigatus tmpL locus. (A) A. fumigatus wild-type tmpL gene locus was replaced by the A. parasiticus pyrG cassette, resulting in Af ΔtmpL mutant locus. The mutated genomic locus of Af ΔtmpL mutant is depicted to show homologous recombination of the replacement cassette. (B) Southern blot analysis of A. fumigatus wild-type strain (CEA10), ΔtmpL mutant (Af ΔtmpL), and reconstituted strain (AftmpL rec). The wild-type strain CEA10 contained a 6.3 kb NcoI fragment. A band shift to about 3.8 kb was detected in Af ΔtmpL strain, indicating that homologous recombination occurred at a single site. The reconstituted strain AftmpL rec showed the same 6.3 kb band to the wild-type and 3.8 kb band to the Af ΔtmpL strain, indicating the Af ΔtmpL mutant has been ectopically complemented by a full-length tmpL gene fragment. The letter N on the genomic locus (A) indicates enzymatic sites for NcoI that were used of genomic DNA digestion. The region used for labeling the hybridization probe is marked with a bar (Probe). (0.06 MB PDF) [file ppat.1000653.s005.pdf]

**A**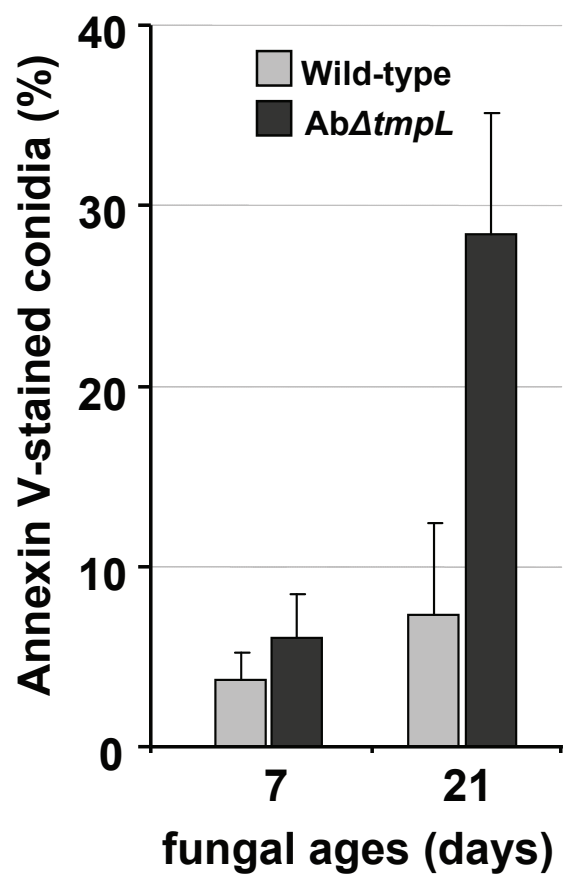**B**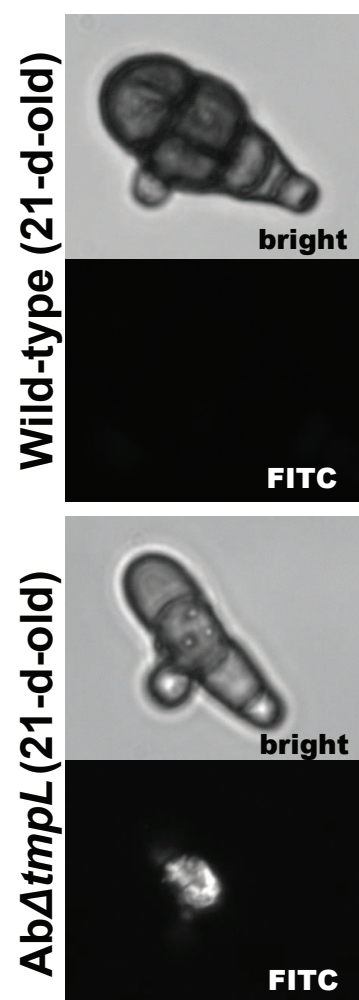

Supplement: Figure S6 — Detection of cell death in A. brassicicola wild-type and ΔtmpL conidia stained with annexin V-FITC. (A) Conidia collected from 7- and 21-day-old fungal colonies grown on solid CM were subjected to annexin V-FITC staining. Percentage of conidia showing fluorescence that are classified as dead cells was measured. Columns and error bars represent average and SD, respectively, of two independent experiments. (B) Representative micrographs showing an annexin V-FITC positive conidial cell of the 21-day-old Ab ΔtmpL mutant, while no staining in the 21-day-old wild-type conidia. (0.25 MB PDF) [file ppat.1000653.s006.pdf]

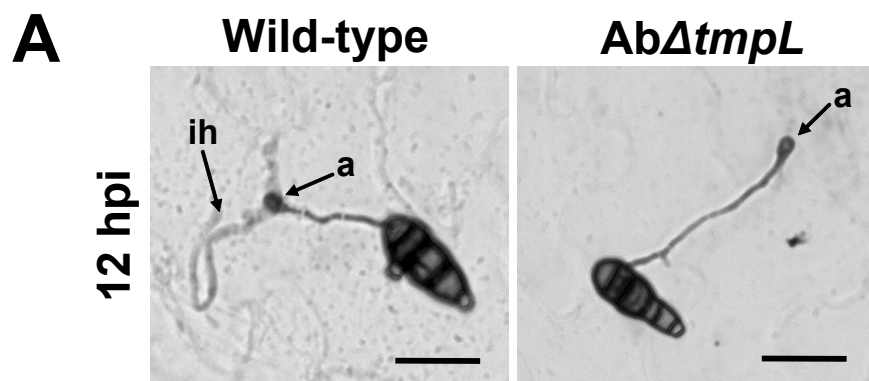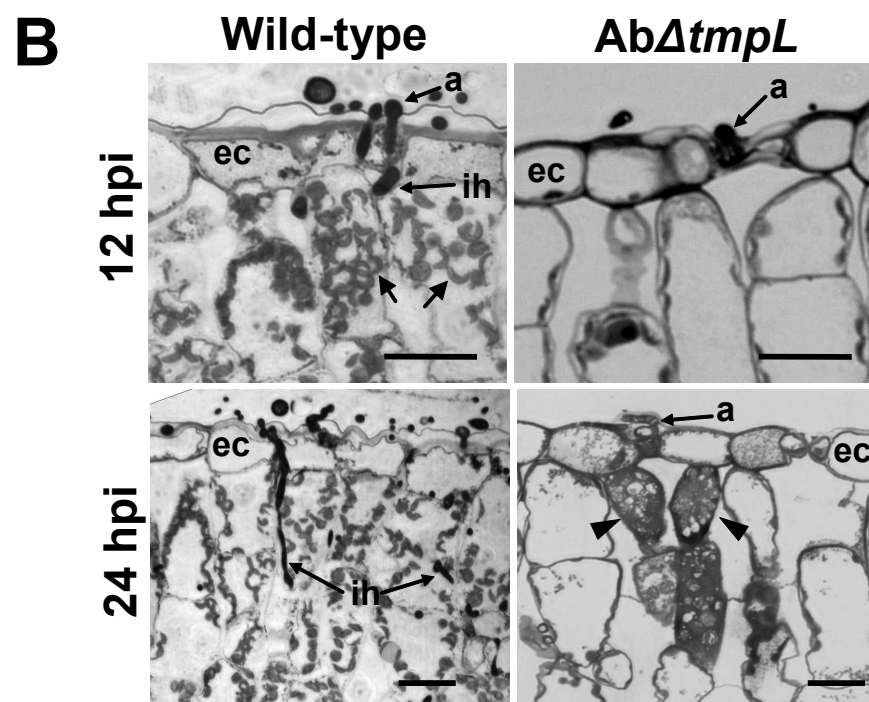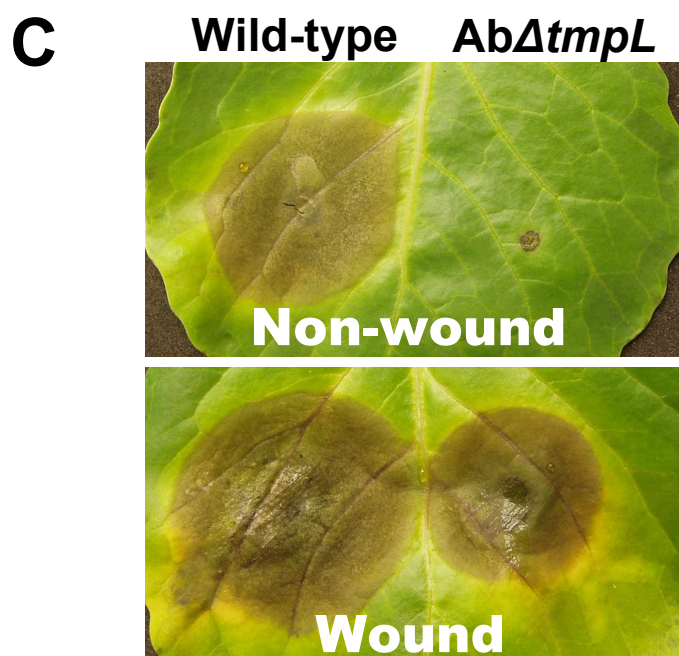

Supplement: Figure S7 — Formation of appressoria and infection hyphae and a virulence assay of wounded and non-wounded green cabbage leaves inoculated with A. brassicicola wild-type and ΔtmpL mutant. (A) Green cabbage cotyledons were used to examine the appressoria and infection hyphae formation of wild-type and Ab ΔtmpL mutant infection. Intracellular infection hyphae of the Ab ΔtmpL mutant were rarely developed inside of the plant epidermal cells, while infection hyphae of the wild-type appressoria were consistently observed. Bars = 20 µm. Abbreviations: a, appressorium; ih, infection hypha. (B) Green cabbage leaves inoculated with wild-type and Ab ΔtmpL mutant were collected at 12 and 24 hpi, embedded epoxy resin, sectioned, and stained with 0.1% toluidine blue O. Due to the massive secretion of fungal enzymes and toxins from the appressoria and infection hyphae of the wild-type, plant tissues around the fungal cells were extensively macerated and degraded and plastids were abnormally inflated (arrows). By contrast, leaf sections inoculated with the Ab ΔtmpL mutant maintained almost intact plant tissue (12 hpi) and plant cells below the infection site showed cell necrosis or callose-deposition-like phenomenon at 24 hpi (arrowheads). Bars = 20 µm. Abbreviations: a, appressorium; ec, epidermal cell; ih, infection hypha. (C) Wounded leaf infection assay of wild-type and Ab ΔtmpL mutant. The upper panel indicates intact (non-wounded) leaf inoculated with the wild-type and Ab ΔtmpL mutant, and the lower panel depicts wounded leaf infection by needle scratching. The Ab ΔtmpL mutant formed larger lesions on wounded leaves compared with the tiny lesions on intact leaves but were still smaller than those resulting from wild-type inoculations on wounded leaves. (0.37 MB PDF) [file ppat.1000653.s007.pdf]
