# Supplementary material for: TmpL, a Transmembrane Protein Required for Intracellular Redox Homeostasis and Virulence in a Plant and an Animal Fungal Pathogen
Source: PLoS Pathog. 2009 Nov 6;5(11):e1000653. doi: 10.1371/journal.ppat.1000653 (PMC2766074; doi:10.1371/journal.ppat.1000653)
Supplement: Figure S2 — Sequence comparison of A. brassicicola TmpL protein with Aspergillus nidulans TmpA protein. The partial amino acid sequence (501–1025) of TmpL is aligned with TmpA protein (GenBank accession no. AAP13095) from A. nidulans using ClustalW2. Identical amino acid residues are indicated by asterisks and similar residues by dots. Predicted transmembrane domains in TmpL and TmpA are underlined. Hypothetical FAD (RLHFD) and NAD(P) (GSGIGP) phosphate-binding domains are indicated with light and dark gray boxes respectively. (0.03 MB PDF) [file ppat.1000653.s002.pdf]

```
TmpL   501  NGKVDKAQLKILAAERNNPVVHVKRLETMHTRSESAFDPLALFTKAMVPRELMRALADVD  560
TmpA     1  -----MSQPEEVIVTRPPTAVCVP-----ESPSETSFVNIEKCIIKDVSELMLPDEPSFD   50
                  :* :  :  .  *   ..* *       .: **::*  :        *.. ::   ...*

TmpL   561  LEKAYPLKSLEDYSLVEKDAPLNIPARLPEPDVSEVTAWLRHRGLIAYRWFLFPIVFANA  620
TmpA    51  LEAQSTARRLISP-------------------------IRYTFLNIYRRLFTLVFLANI   84
            **    .  :  *  .                        :*:   *   **  ::   :.:**

TmpL   621  GVACWLLYRYMQGSKYPLSPTATATASNLCAAILIRSEPVINLLFLVCSSVPTSTPLWIR  680
TmpA    85  GVFVYVMVADR-----KLLALVNAAAANLLACGLARQPLVVNTIFFTVCSIPRSAPLWLR  139
            **   :::              *  .  ..*:*:** *.  *  *.   *:*  :*:.  .*:* *:***:*

TmpL   681  RIFAKVFHIGGIHVGCAIAAVMWFVIFTVGASLEMARNDDERERSVLPAALSYLTLLLLI  740
TmpA   140  RIASKVYHYGGVHSGCGVASLIWYLGFIGEFSRQYWSGSSS-PFSAAPIVLAYIILVLLL  198
            ** :**:* **:* **.:*:::*:: *     * :     ....    *.  *  .*:*: *:**:

TmpL   741  AMTSLSHPVFRNRHHDLWEALHRFGGWTVLILYWVLVGLSTKDLAHGRTLSTSEAYLQTP  800
TmpA   199  AIIIVAYPTFRFKRHDYFELTHRFSGWLIVALFVILLMVFVDEASAAEGKPMGRFLIELP  258
            *:   :::*.** ::** :*   ***.** :: *: :*: :  ..: :  ..   .  ..   :: *

TmpL   801  SLWLITAATCAIIFPWLFLRRVPVRPEVLSSHAVRLHFDSEVTP-GKGVRLAQHPLRDWH  859
TmpA   259  AFWFLMLVVLAIIHPWLLLRKVKVTPEYLSPHAVRLHFSHTTTTFGKGIQLSKHPLQDWH  318
            ::*::   ..  ***.***:**:* * ** **.*******.   .*.  ***::*::***:***

TmpL   860  GFATITNAP-GGKGHSVIVSRAGDFTGNMIDTAPTHIWRRGIPTSGVLRIATLFKSVVVV  918
TmpA   319  GFATFPDVDRDGKSFSSLVSKAGDWTAATIKDQPTHLWKRGVLIYGFAYAMRVYKRVVVV  378
            ****:.:.   .**..* :**:***:*.   *.  ***:*:**:    *.      ::* ****

TmpL   919  ATGSGIGPCLSIFPYR-HVAMRVLWTAPNHETTFGKAIVDAVRRKDPRAVLYNTRTSGKP  977
TmpA   379  TTGSGIGPCLSFLGDENRPSLRVIWQTRAPKRTYGKEVLNLVGRMDPNPVIIDTNSSGRL  438
            :**********::   .  :  ::**:* :     : *:** ::: * * **..*: :*.:**:

TmpL   978  DMSLLAYRVYKESDAEAVLVISNKRFTQQIVFDMEKRGIPAYGAIFDS  1025
TmpA   439  DMVPVIRQIAREHDAEAICVISNPFVTKKVVYELESMGIPAYGPIFDS   486
            **   :   :: :* ****: ****   .*:::*:::*. ******.****
```
