# Supplementary material for: TmpL, a Transmembrane Protein Required for Intracellular Redox Homeostasis and Virulence in a Plant and an Animal Fungal Pathogen
Source: PLoS Pathog. 2009 Nov 6;5(11):e1000653. doi: 10.1371/journal.ppat.1000653 (PMC2766074; doi:10.1371/journal.ppat.1000653)
Supplement: Table S1 — Primer sequences used in this study. (0.02 MB PDF) [file ppat.1000653.s008.pdf]

## Table S1. Primer sequences used in this study

| OLIGO | SEQUENCE 5´ TO 3´ |
|---|---|
| *Alternaria brassicicola* **Primers** | |
| **ATM1R1** | TCAGCAGAGCATGAAGTTCG |
| **ATM1R2** | ATCAGTTAACGTCGACCTCGAATTGCGAGGGTAATTGACG |
| **ATM1R3** | CGTCAATTACCCTCGCAATTCGAGGTCGACGTTAACTGAT |
| **ATM1R4** | TCGTTTGGCACATTACCTCACGTCGACGTTAACTGGTTCC |
| **ATM1R5** | GGAACCAGTTAACGTCGACGTGAGGTAATGTGCCAAACGA |
| **ATM1R6** | CACAGGCACAATTCACAAGG |
| **ATM1comF** | TCTAGGCTAGCAGGCAGCTC |
| **ATM1comR** | CGAACTTCATGCTCTGCTGA |
| **PNRcomF** | GAGCGGAGACATGTGAGACC |
| **PNRcomR** | TCATTCTAGCTTGCGGTCCT |
| **ATM1GFP1** | TTTCACATTGGCGGCATACA |
| **ATM1GFP2-GA** | ACCATGGCACCGGCTCCAGCGCCTGCACCAGCTCCTGAATCGAAAATAGCGCCATAG |
| **ATM1GFP3-6GA** | ATTCAGGAGCTGGTGCAGGCGCTGGAGCCGGTGCCATGGTGAGCAAGGGCGAGGA |
| **ATM1GFP4** | GTTCCCGGTCGGCATCTACT |
| **A1AdeGFP1** | CTGGTAGGCTTGCGAGAGTC |
| **A1AdeGFP2-GA** | ACCATGGCACCGGCTCCAGCGCCTGCACCAGCTCCAGCTGTCACCTCTGAAACATCC |
| **A1AdeGFP3-GA** | CAGCTGGAGCTGGTGCAGGCGCTGGAGCCGGTGCCATGGTGAGCAAGGGCGAGGA |
| **A1AdeGFP4** | GTTCCCGGTCGGCATCTACT |
| **A1TmGFP1** | CTCAACATACCGGCCAGACT |
| **A1TmGFP2** | ACCATGGCACCGGCTCCAGCGCCTGCACCAGCTCCGGCGTGCGAGGATAAGAC |
| **A1TmGFP3** | ACGCCGGAGCTGGTGCAGGCGCTGGAGCCGGTGCCATGGTGAGCAAGGGCGAGGA |
| **A1TmGFP4** | GTTCCCGGTCGGCATCTACT |
| **ToxAFor** | GCGTAGAGTTTGAGCGATCC |
| **ToxA-DsRedRev** | GTTCTCGGAGGAGGCCATATCACGCTTGAACTTTTTGG |
| **DsRed-ToxAFor** | CCAAAAAGTTCAAGCGTGATATGGCCTCCTCCGAGAAC |

| | |
|---|---|
| **DsRed-AbHex1Rev** | CTCGTTGTCGTAATATCCCATGGCTCCTGCAGCCAGGAACAGGTGGTGGCG |
| **AbHex1-DsRedFor** | CGCCACCACCTGTTCCTGGCTGCAGGAGCCATGGGATATTACGACAACGAG |
| **AbHex1Rev** | CGAACGTTTGTACAATCACCTG |
| **DsRedPTS1For** | ACCTTGGAATGCATGGAGGA |
| **DsRedPTS1Rev** | GAATTCGATATCAAGCTTCTACAGCTTCGACAGGAACAGGTGGTGGCG |
| **DsRedPTS1NATFor** | CGCCACCACCTGTTCCTGTCGAAGCTGTAGAAGCTTGATATCGAATTC |
| **DsRedPTS1NATRev** | TCATTCTAGCTTGCGGTCCT |
| **pex14KOFor** | CAGCCTACCTTCACCTCCAC |
| **pex14KORev** | GGTCTCACATGTCTCCGCTCTTGTTGTCCTCGCCTCTTCT |
| **pex14HygFor** | AGAAGAGGCGAGGACAACAAGAGCGGAGACATGTGAGACC |
| **pex14HygRev** | TCATTCTAGCTTGCGGTCCT |
| **PromoYap1For** | GCTAGGTTGCCGCATTGGAC |
| **PromoYap1Rev** | TCCTCGCCCTTGCTCACCATTGCAAGTGCGCTTGCGGGG |
| **GFPYap1For** | CCCCGCAAGCGCACTTGCAATGGTGAGCAAGGGCGAGGA |
| **GFPYap1Rev** | AAGTCGTTAGTAGTTCCTGCCATGGCTCCTGCAGCCTTGTACAGCTCGTCCATGC |
| **Yap1For** | GCATGGACGAGCTGTACAAGGCTGCAGGAGCCATGGCAGGAACTACTAACGACTT |
| **Yap1Rev** | GACCGCAAGCTAGAATGATTACCCAAGCAGTTTGTCCT |
| **Yap1NATFor** | AGGACAAACTGCTTGGGTAATCATTCTAGCTTGCGGTC |
| **Yap1NATRev** | CTGCAGGCCGCTCAGGGGCAGG |
| **TxAFor** | AGGAGTTCTGTACGCGCAAT |
| **ToxAYap1Rev** | AAGTCGTTAGTAGTTCCTGCCATGGCCTATATTCATTCATTGTCAGC |
| **Yap1overFor** | GCTGACAATGAATGAATATAGGCCATGGCAGGAACTACTAACGACTT |
| **ATM1-expFor** | TCTGAAGTAACACCAGGCAAGGGT |
| **ATM1-expRev** | TCCATATATGTGTCGGCGCGGTAT |
| **YAP1- expFor** | TCTGAAGTAACACCAGGCAAGGGT |
| **YAP1- expRev** | TCCATATATGTGTCGGCGCGGTAT |
| **SKN7-expFor** | ACATCATCATGCCGAACCTTGACG |
| **SKN7-expRev** | ATGGAGATGTCGTCGCTTCGGATA |
| **CTT1- expFor** | TCTGAAGTAACACCAGGCAAGGGT |

| | |
|---|---|
| **CTT1- expRev** | TCCATATATGTGTCGGCGCGGTAT |
| **SOD1- expFor** | TCTGAAGTAACACCAGGCAAGGGT |
| **SOD1- expRev** | TCCATATATGTGTCGGCGCGGTAT |
| **GSH1- expFor** | TCTGAAGTAACACCAGGCAAGGGT |
| **GSH1- expRev** | TCCATATATGTGTCGGCGCGGTAT |
| **GSH2- expFor** | TCTGAAGTAACACCAGGCAAGGGT |
| **GSH2- expRev** | TCCATATATGTGTCGGCGCGGTAT |
| **TRX2- expFor** | TCTGAAGTAACACCAGGCAAGGGT |
| **TRX2- expRev** | TCCATATATGTGTCGGCGCGGTAT |
| **GPX1- expFor** | TCTGAAGTAACACCAGGCAAGGGT |
| **GPX1- expRev** | TCCATATATGTGTCGGCGCGGTAT |
| **AbGAPDH-For** | TGGAAAGAACCTCGATGTCGGACT |
| **AbGAPDH-Rev** | ACGTTGTTGAGTCCACTGGTGTCT |
| **A1fn_ExpKpnFor** | TGGTACCGTCCCTGTTCGGCCCGAG |
| **A1fn_ExpHndRev** | TAAGCTTTCTGGCTGCCCTATCATGTTTCA |
| | |
| ***Aspergillus fumigatus* Primers** | |
| **RAC21** | TGGCGACCACACCCGTCCTGTG |
| **RAC22** | TAGGGTACCTGTCCGCGCGGGG |
| **RAC39** | CCGTCGACTAAGCACGAGCACGAATCAC |
| **RAC41** | ATTCTAGAAATGATCGGCTTCGCATATC |
| **RAC109** | ACTCAGCTACATGCTGCCTACGTT |
| **RAC110** | AGCTGATTACGAAAGCTCTCGCCT |
| **RAC325** | ATCCTTGAAGCTGTCCCTGATGGT |
| **RAC326** | TCCGGCTCGTATGTTGTGTGGAAT |
| **RAC351** | ATGGGTGAGCATTGGAGGACTGAA |
| **RAC352** | ACCTGATCATCATTGCGTCCCAGA |
| **RAC357** | TTGCGGCCGCAACTCAGCTACATGCTGCCTACGTT |
